# Sexual dimorphisms of mRNA and miRNA in human/murine heart disease

**DOI:** 10.1371/journal.pone.0177988

**Published:** 2017-07-13

**Authors:** Masato Tsuji, Takanori Kawasaki, Takeru Matsuda, Tomio Arai, Satoshi Gojo, Jun K. Takeuchi

**Affiliations:** 1 Division of Bio-informational Pharmacology, Medical Research Institute, Tokyo Medical Dental University, Tokyo, Japan; 2 Department of Biological Sciences, Graduate School of Science, The University of Tokyo, Tokyo, Japan; 3 Department of Cardiovascular Medicine, Kyoto Prefectural University of Medicine, Kyoto, Japan; 4 Department of Mathematical Informatics, Graduate School of Information Science and Technology, The University of Tokyo, Tokyo, Japan; 5 Department of Pathology, Tokyo Metropolitan Geriatric Hospital and Institute of Gerontology, Tokyo, Japan; Queen's University Belfast, UNITED KINGDOM

## Abstract

**Background:**

Sexual dimorphisms are well recognized in various cardiac diseases such as ischemic cardiomyopathy (ICM), hypertrophic cardiomyopathy (HCM) and dilated cardiomyopathy (DCM). Thorough understanding of the underlying genetic programs is crucial to optimize treatment strategies specified for each gender. By performing meta-analysis and microarray analysis, we sought to comprehensively characterize the sexual dimorphisms in the healthy and diseased heart at the level of both mRNA and miRNA transcriptome.

**Results:**

Existing mRNA microarray data of both mouse and human heart were integrated, identifying dozens/ hundreds of sexually dimorphic genes in healthy heart, ICM, HCM, and DCM. These sexually dimorphic genes overrepresented gene ontologies (GOs) important for cardiac homeostasis. Further, microarray of miRNA, isolated from mouse sham left ventricle (LV) (n = 6 & n = 5 for male & female) and chronic MI LV (n = 19 & n = 19) and from human normal LV (n = 6 & n = 6) and ICM LV (n = 4 & n = 5), was conducted. This revealed that 13 mouse miRNAs are sexually dimorphic in MI and 6 in normal heart. In human, 3 miRNAs were sexually dimorphic in ICM and 15 in normal heart. These data revealed miRNA-mRNA networks that operate in a sexually-biased fashion.

**Conclusions:**

mRNA and miRNA transcriptome of normal and disease heart show significant sex differences, which might impact the cardiac homeostasis. Together this study provides the first comprehensive picture of the genome-wide program underlying the heart sexual dimorphisms, laying the foundation for gender specific treatment strategies.

## Background

Sexual dimorphisms are well recognized in various cardiac diseases [1] [2]. Ischemic cardiomyopathy (ICM) including myocardial infarction (MI) develops later in women, but once established, it contributes more persistent symptoms and higher mortality than in men [3–13]. Hypertrophic cardiomyopathy (HCM) reportedly shows similar trends [14–18]. On the contrary, more prevalent in men is dilated cardiomyopathy (DCM), both familial and myocarditis-induced [19–28]. Importantly, similar observations have been reported in rodent models of ICM [29,30], HCM [31–35], and DCM[36–39], offering powerful models to elucidate the underlying molecular mechanisms. However, study results focusing on the effect of sex hormones so far have been conflicting [31–33,36,38,40–43]. Therefore, the whole picture of sexual dimorphism in the heart remains unclear.

Several research teams thus investigated the mRNA transcriptome in mouse MI [44], HCM [31,32,45], DCM[39], and human DCM [46,47], and found sexually dimorphic genes. However, whether or not such sex differences also exist in human ICM and HCM is still unknown. In addition, no study has investigated the sex difference of cardiac disease at the level of miRNAs, important players in cardiac functions and diseases [48]. Comprehensive understanding of the mRNA- and miRNA-level genetic programs underlying the heart sexual dimorphisms will expectedly improve clinical outcome by facilitating the development of gender specific treatment strategies.

Here, by conducting meta-analysis of mRNA transcriptome and performing miRNA microarray analysis of mouse/ human disease samples, we set out to characterize the heart sexual dimorphisms at the level of both mRNA and miRNA transcriptome. mRNA meta-analysis identified dozens/ hundreds of sexually dimorphic genes in ICM, HCM, DCM, and normal heart. These genes over-represented GOs important for cardiac homeostasis, suggesting the functional significance of their sex difference. Next we investigated the miRNA in ICM and normal heart and found significant sex difference. Computational analyses suggest that these sexually dimorphic mRNAs and miRNAs form sex-specific miRNA-mRNA networks. Together these data provide the first comprehensive picture of the genome-wide program underlying the heart sexual dimorphisms, laying the foundation for the gender specific treatment strategies.

## Methods

An expanded Methods section is available in S1 Methods. All microarray data have been submitted to the National Center for Biotechnology Information gene expression and hybridization array data repository (GSE76604).

### Myocardial infarction modeling

The left anterior descending (LAD) coronary artery of mice aged 10 weeks was surgically ligated to create extensive MI. The ventricular septum of the areas at risk of ischemia was sampled on post-operative day 28.

### Patient selection and tissue collection

Human tissue samples, acquired during post-mortem examination and frozen in liquid nitrogen, were provided by the Department of Pathology, Tokyo Metropolitan Geriatric Hospital. Autopsy and medical research were performed with written consent by the families under the Act of Postmortem Examination. This work was approved by the ethical committee of the Tokyo Metropolitan Geriatric Hospital (no.240208). Age- and sex-matched cohorts were selected to compare healthy hearts to those with post-MI LV remodeling. Border zone for myocardial infarction was sampled for microarray analysis (S1 Methods).

### RNA library preparation, microarray, and data processing

Total RNA was extracted from samples using Sepasol (Sepasol-RNA I super G, nakalai tesque, Japan), and microarray analysis was performed using Affymetrix GeneChip® miRNA 3.0 Arrays.

### Public microarray data integration

Relevant studies were searched in the NCBI Gene Expression Omnibus (GEO)[49] and their raw data were downloaded and processed in R[50] according to each array platform. We used 4 mouse studies (GSE23294[44], GSE18224[31,32], GSE6970[45], GSE35182[39]) and 6 human studies (GSE57338[51], GSE29819[52], GSE22253[53], GSE26887[54], GSE52601[55], GSE36961 (Hebl VB, Bos JM, Oberg AL, Sun Z, Herman DS, Teekakirikul P, Seidman JG, Seidman CE, dos Remedios CG, Schaff HV, Dearani JA, Ommen SR, Brozovich FV, Ackerman MJ, unpublished data, [2012])) reporting the mRNA transcriptome of both genders belonging to either normal/ ICM/ HCM/ DCM (Table 1). The cross-platform normalization was performed using COMBAT method[56].

**Table 1:** Characteristics of the microarray data used in this study.

GSE	Disease	Age (s.d.) *	Sample Size (M_F)	Platform name	d.a.o†	Procedure
**Mouse**						
GSE23294	Normal	12~15	10	Illumina MouseWG-6 v2.0 expression beadchip	3	Sham
			(5_5)			
GSE23294	AMI	12~15	10	Illumina MouseWG-6 v2.0 expression beadchip	3	LAD ligation
			(5_5)			
GSE18224	Normal	10	8	Affymetrix Mouse Genome 430 2.0 Array	63	Sham
			(4_4)			
GSE18224	HCM	10	8	Affymetrix Mouse Genome 430 2.0 Array	63	TAC
			(4_4)			
GSE6970	Normal	12~14	8	Affymetrix Mouse Expression 430A Array	16	Sham
			(4_4)			
GSE6970	HCM	12~14	8	Affymetrix Mouse Expression 430A Array	16	TAC
			(4_4)			
GSE35182	Normal	6~8	6	Affymetrix Mouse Gene 1.0 ST Array	90	Sham
			(3_3)			
GSE35182	DCM	6~8	6	Affymetrix Mouse Gene 1.0 ST Array	90	CVB3-induced myocarditis
			(3_3)			
**Human**						
GSE57338	Normal	49.4 (15.0)	136	Affymetrix Human Exon ST1.1 arrays		
			(73_63)			
GSE57338	ICM	59.1 (7.4)	95	Affymetrix Human Exon ST1.1 arrays		
			(81_14)			
GSE57338	DCM	51.2 (14.0)	82	Affymetrix Human Exon ST1.1 arrays		
			(63_19)			
GSE29819	Normal	45.2 (18.8)	5	Affymetrix Human Genome U133 Plus 2.0 Array		
			(3_2)			
GSE29819	DCM	57.6 (6.1)	7	Affymetrix Human Genome U133 Plus 2.0 Array		
			(3_4)			
GSE22253	Normal	47.9 (12.6)	107	Affymetrix Human Gene 1.0 ST Array		
			(55_52)			
GSE26887	Normal	48.4 (2.6)	5	Affymetrix Human Gene 1.0 ST Array		
			(2_3)			
GSE26887	ICM	62.2 (12.1)	19	Affymetrix Human Gene 1.0 ST Array		
			(18_1)			
GSE52601	Normal	52 (10.6)	3	Illumina HumanHT-12 V4.0 expression beadchip		
			(3_0)			
GSE52601	ICM	67 (2.6)	4	Illumina HumanHT-12 V4.0 expression beadchip		
			(3_1)			
GSE52601	DCM	56 (9.4)	4	Illumina HumanHT-12 V4.0 expression beadchip		
			(3_1)			
GSE36961	Normal	37.2 (15.2)	39	Illumina HumanHT-12 V3.0 expression beadchip		
			(19_20)			
GSE36961	HCM	46.6 (18.8)	106	Illumina HumanHT-12 V3.0 expression beadchip		
			(54_52)			

* Unit is week for mouse and year for human

†Days after onset

### Differential expression analysis

The normalized data were analyzed using R and the Bioconductor limma package[57]. All p-values were adjusted for false discovery rate correction (FDR < 0.05). Since cardiac sex difference presumably reflects the mild biases in transcriptome-wide gene expression, 1.2 fold change was considered biologically meaningful. Hence our detection criteria were: FDR < 0.05 and fold change > 1.2.

### Data analysis

Statistical analyses were conducted in R unless specified otherwise. p < 0.05 was considered significant.

## Results

### Study design

**Fig 1:**
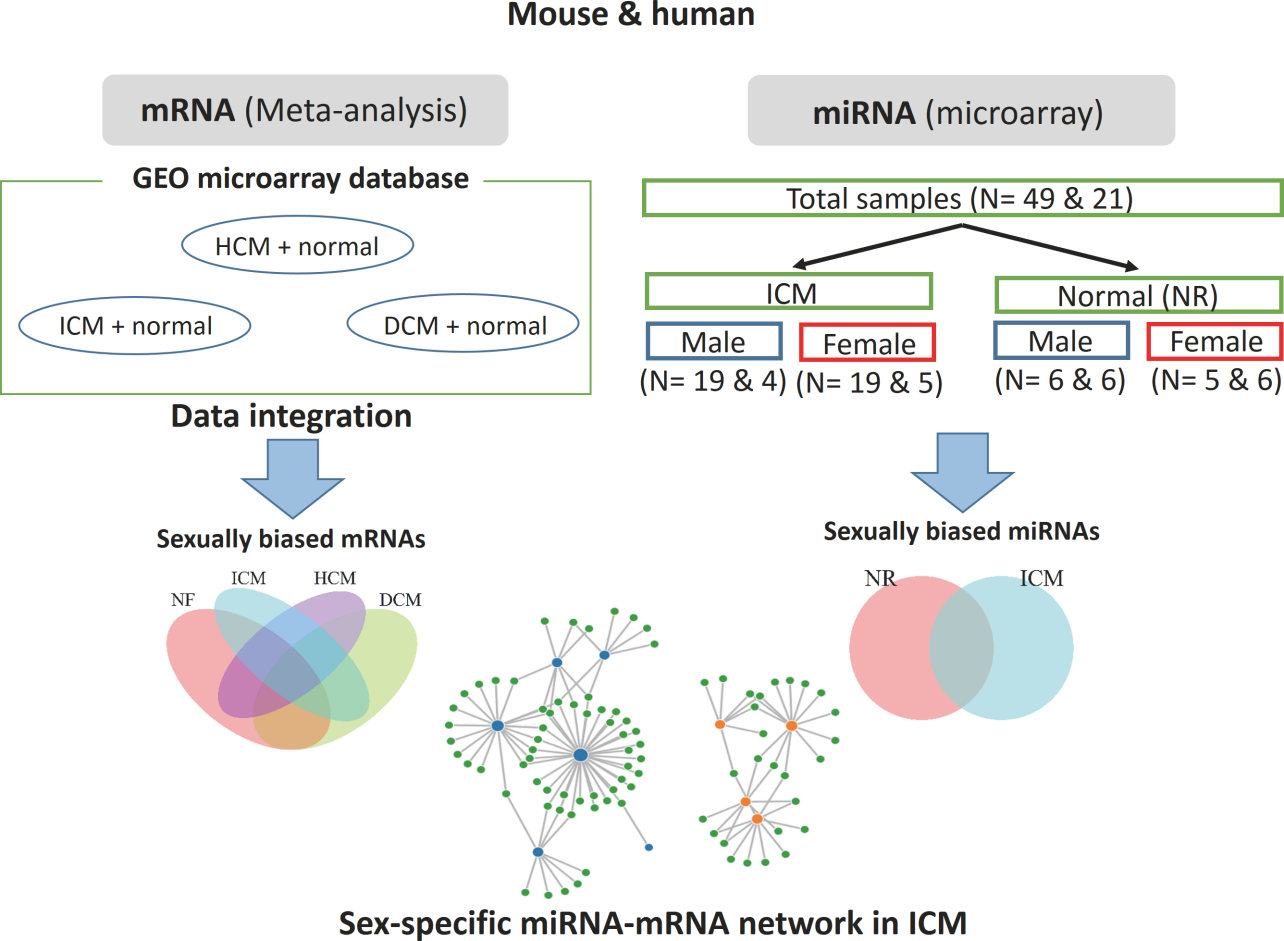
Workflow of this study. For mRNA, public microarray data were integrated with cross-platform normalization. The integrated mRNA data included normal (NR) and ICM samples as well as HCM and DCM. For miRNA data, miRNA microarray analysis was conducted using 49 mouse samples and 21 human samples. These samples included normal and ICM samples. Sample sizes are indicated in the bracket (mouse & human). In both mRNA and miRNA microarray analyses, male and female expression levels were compared within each disease group. Finally, mRNA and miRNA data from ICM samples were used to identify sexually biased miRNA-mRNA networks.

In this study, we sought to characterize the sex difference in mRNA and miRNA transcriptome (Fig 1). Since GEO database offered several mRNA microarray data of both genders of disease heart, we conducted meta-analysis to assess the mRNA sex difference. For miRNA data, for which such data was not available, we performed microarray analysis.

### Integrating public microarray data reveals significant sex difference of mRNA expression in normal/ disease heart

**Fig 2:**
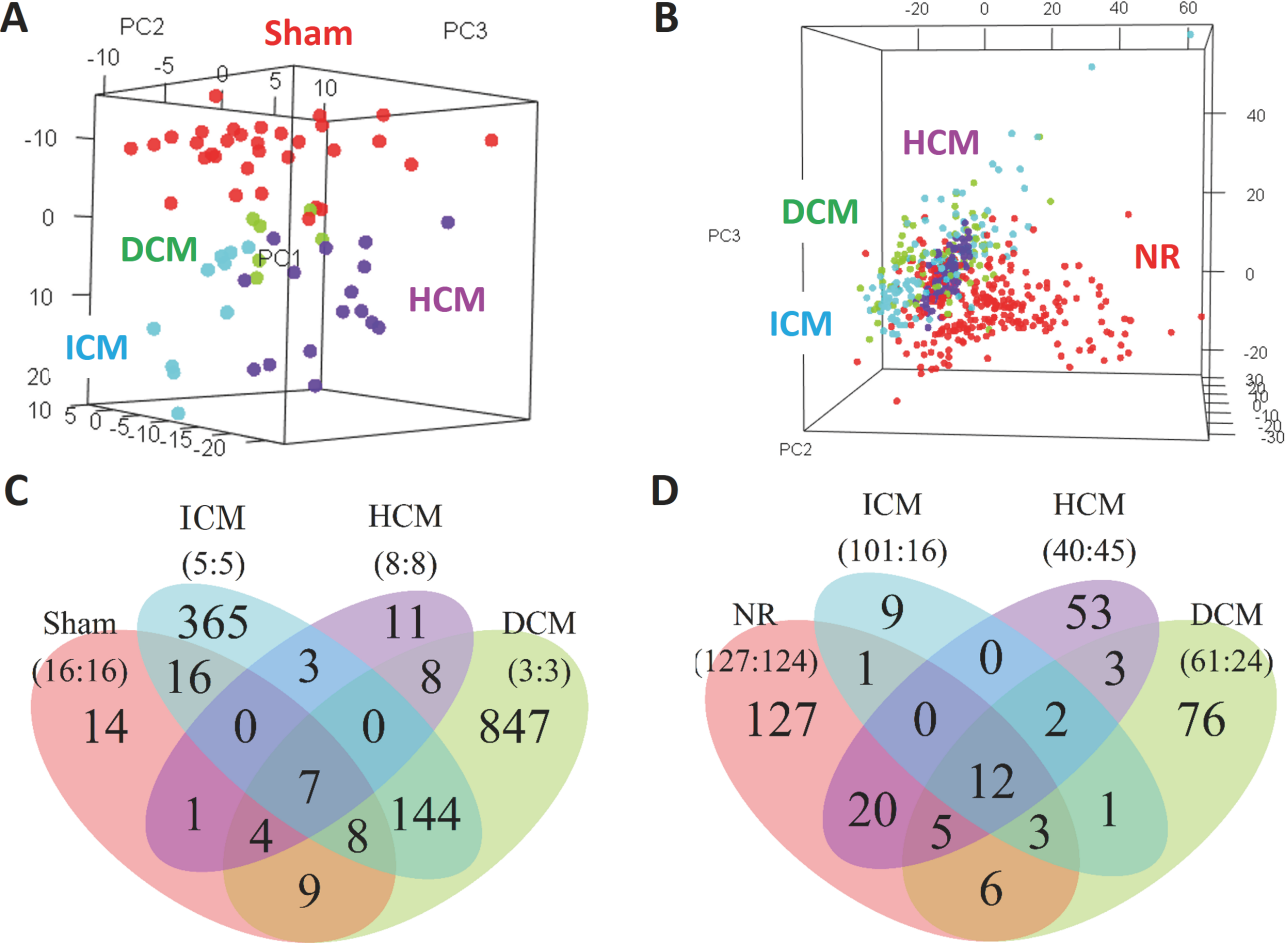
mRNA array data integration. (A, B) PCA of the integrated mouse (A) and human (B) data. Samples of the same health condition are grouped together, indicating that our cross-platform normalization procedure effectively removed the batch effect.(C, D), Venn’s diagram of the results of male-female comparison in mouse (C) and human (D). Dozens/ hundreds of genes were found to be sexually biased in each health condition Sample sizes are shown in bracket (male: female).

First, we asked if disease heart shows sex difference in mRNA expression. From GEO database, we found 4 mouse studies and 6 human studies that reported the transcriptome of the normal/ ICM/ HCM/ DCM (Table 1). The sample size of mouse and human data totaled 64 and 616, respectively. These data were each pre-processed according to their array platform and then integrated with COMBAT cross-platform normalization method[56]. Principal component analysis (PCA) confirmed that the cross-platform normalization effectively removed the batch effect–the noise arising from the fact that each data is generated by different labs and platforms (Fig 2, S1 Fig).

To assess whether sex difference exists in our combined meta-data, we checked if sex can be discriminated on the basis of the transcriptomic principal components (PCs). PCs are the products of PCA, a popular dimensionality reduction technique. In this analysis, the expression values of all genes are linearly combined to construct a small set of new variables so that they best retain the transcriptome information. When we performed machine-learning discrimination using PCs, male and female were discriminated almost perfectly (S2–S4 Figs), indicating the existence of sex difference. Likewise, health conditions were also effectively discriminated, confirming that different diseases acquire different transcriptome (S5 Fig).

Consistently, we identified a number of genes sexually biased in all the health conditions (Fig 2C and 2D, S1 and S2 Tables). As expected, these genes were enriched on sex chromosomes among several other chromosomes, possibly accounting for their sex difference (S6 Fig). The overrepresented GOs included angiogenesis, cardiac muscle growth, regulation of heart contraction, and response to wounding (S3 Table), suggesting the functional importance of these sexually dimorphic genes in heart disease. These data are consistent with previous reports [32,58–60].

### miRNA array sample characteristics

Next we asked if the similar sex difference also exists in miRNA expression. To this end we conducted miRNA microarray analysis using ICM and normal heart samples of both mouse and human. Murine ICM model samples were prepared by surgically ligating the LAD coronary artery and were sacrificed 1 month later. The hearts were macroscopically validated to exhibit the LV free wall thinning and dilatation (S7 Fig). Human RNA samples were obtained during post-mortem examination. Patient characteristics are summarized in Table 2.

**Table 2:** Clinical characteristics of the study subjects.

	Normal	ICM
Sample number	12	9
Age, mean (s.d.)	79.83 (6.76)	79.22 (4.81)
Male, ratio	50	44.44
BMI, kg/m2 mean (s.d.)	17.04 (7.03)	19.5 (6.93)
Medical history, ratio		
AS	0	0.11
LVH	0	0.56
Hypertension	0.42	0.89
DM	0	0.67
Smoking	0.55	0.43

ICM: Ischemic cardiomyopathy, BMI: body mass index, AS: aortic stenosis, LVC: left ventricular hypertrophy, DM: Diabetes mellitus

### Sex difference of miRNA expression in normal heart and ICM

**Fig 3:**
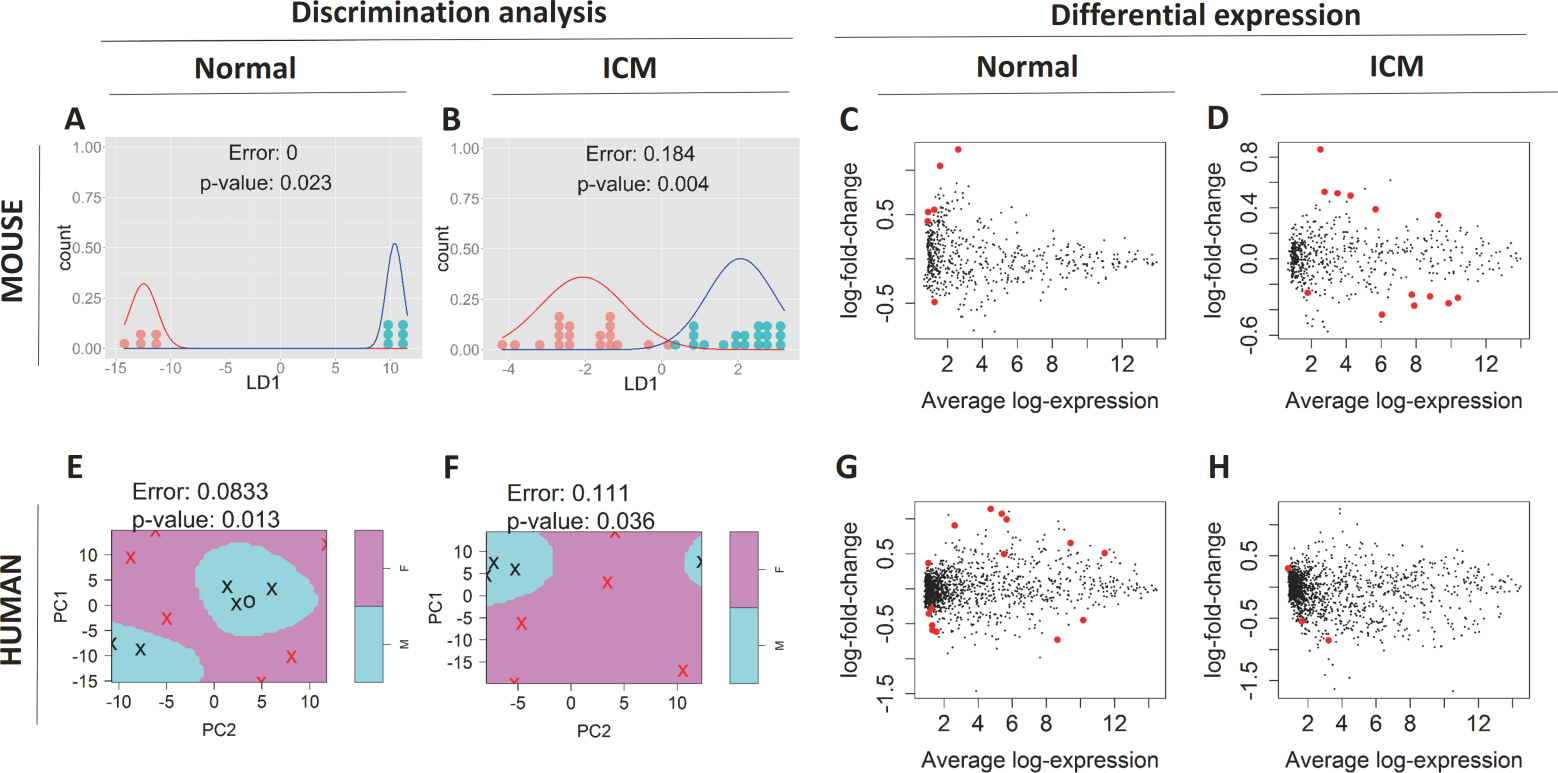
miRNA microarray comparison result. (A, B) Sex discrimination of mouse miRNA array data of sham (A) and ICM (B) by LDA based on PCs. Each dot indicates one mouse sample. Shown curves are fitted normal distributions. Red: female, blue: male. Male and female samples are grouped together on the number line of a linear discriminant (LD1), and thus are well discriminated with p-value < 0.05 (C, D) MA plot of sex comparison in sham (C) and ICM (D) mouse data. Each dot indicates one miRNA. Sexually dimorphic miRNAs are colored red. (E, F) Sex discrimination of human miRNA array data of normal (E) and ICM (F) by SVM based on PCs. Crosses indicate data used as support vectors and circles the other. Red crosses/ circles indicate female samples and black crosses/ circles indicate male. Pink-/ blue- shaded regions indicates female/ male regions determined by SVM, respectively. Male and female samples are well discriminated with p-value < 0.05. (G, H) MA plot of sex comparison in normal (G) and ICM (H) patient data. The LOOCV error rates and their associated p-values are shown in (A), (B), (E), (F).

We profiled expression of 1088 mouse mature miRNAs using a high-throughput Affymetrix platform. 592 miRNAs were expressed above detection threshold in at least one condition and were used for subsequent analyses. We confirmed the reliability of our expression profiling by qPCR (S8 Fig). Machine-learning discrimination on the basis of PCs discriminated male and female almost perfectly, indicating the existence of sex difference (Fig 3A and 3B, S9 and S10 Figs). Likewise, health conditions were discriminated effectively, confirming the change in transcriptome post MI (S11 Fig). Differential expression analysis identified 6 miRNAs as sexually biased in normal heart, and 13 in MI (Fig 3C and 3D, Table 3). As expected, substantially larger number of miRNAs were differentially expressed between normal and MI compared with sexually biased miRNAs (S4 Table). No miRNA showed sex difference both in normal and MI heart, implying that the sex difference in miRNA changes when the heart suffers MI.

**Table 3:** Sexually dimorphic miRNAs.

miRNA	logFC (M/F)	FDR
Mouse Sham		
mmu-miR-190a-3p	1.23154	2.15E-02
mmu-miR-509-5p	1.04725	2.15E-02
mmu-miR-743b-3p	0.52788	2.15E-02
mmu-miR-669k-3p	0.55455	4.36E-02
mmu-miR-1b-3p	0.42497	4.72E-02
mmu-miR-218-5p	-0.4849	4.88E-02
Mouse MI		
mmu-miR-505-5p	0.85863	4.14E-04
mmu-miR-744-5p	0.38993	1.13E-02
mmu-miR-210-3p	-0.3669	1.13E-02
mmu-miR-30e-5p	-0.3482	1.19E-02
mmu-miR-30b-5p	-0.306	1.56E-02
mmu-miR-29b-3p	-0.4383	2.06E-02
mmu-miR-19b-3p	-0.2945	2.06E-02
mmu-miR-193a-5p	0.49665	2.06E-02
mmu-miR-23a-5p	0.52715	0.02063
mmu-miR-142a-5p	-0.2667	0.02589
mmu-miR-664-5p	0.51445	0.03407
mmu-miR-133a-5p	-0.282	0.03755
mmu-miR-214-3p	0.34282	0.04969
Human normal		
hsa-miR-558	-0.5248	4.41E-03
hsa-miR-3187-5p	0.90705	5.25E-03
hsa-miR-365a-3p	-0.6169	5.25E-03
hsa-miR-4669	1.07475	5.25E-03
hsa-miR-1261	0.36894	5.25E-03
hsa-miR-193b-3p	-0.4488	5.98E-03
hsa-miR-4735-3p	-0.2836	5.98E-03
hsa-miR-148a-5p	-0.3558	5.98E-03
hsa-miR-181c-5p	0.9944	0.00598
hsa-miR-4284	-0.7285	0.007
hsa-miR-150-3p	1.14222	0.00708
hsa-miR-4263	-0.5913	0.00708
hsa-miR-4745-5p	0.65563	0.01521
hsa-miR-4634	0.49747	0.04076
hsa-miR-4516	0.51108	0.04189
Human ICM		
hsa-miR-3615	-0.8528	2.97E-02
hsa-miR-4423-5p	0.29816	2.97E-02
hsa-miR-4709-3p	-0.5457	4.66E-02

Next we profiled expression of 1725 human mature miRNAs using the same Affymetrix platform. 1559 miRNAs were expressed above detection threshold in at least one condition. In accordance with mouse, male and female were discriminated almost perfectly on the basis of PCs, indicating that the existence of sex difference is conserved in human heart as well (Fig 3E and 3F, S9 and S10 Figs). 15 and 3 miRNAs were detected as sexually biased in normal heart and ICM, respectively (Fig 3G and 3H, Table 3). Again, no miRNA showed sex difference both in normal and ICM heart.

Interestingly, in mouse nor human, the sexually biased miRNAs were not enriched on sex chromosomes (S12 Fig), implying a dedicated genetic program that creates the sexual biases of these miRNAs.

### Sexually biased miRNAs in ICM are predicted to target cardiomyopathy pathways and are rich in known regulators of cardiac diseases

To gain insight into the sexually dimorphic miRNAs’ functionality in ICM, we performed the target GO/ pathway enrichment analysis on the basis of the target prediction analysis. DIANA microT-CDS software[61,62] and DAVID GO/ pathway enrichment analysis software[63,64] were used for this analysis. DAVID GO/ pathway enrichment analysis was performed with default parameters to rule out the authors’ arbitrariness. The result revealed that the sexually dimorphic miRNAs of ICM and normal heart preferentially target the cardiomyopathy pathways and GOs such as cardiac muscle growth, angiogenesis, apoptosis, and cation transport, important GOs for heart homeostasis (S5 Table). This raises the possibility that these miRNAs account for the sex difference in susceptibility and prognosis of ICM. Consistently, as many as 8 out of 13 sexually biased miRNAs in mouse MI were known to regulate cardiac diseases[65–80] (S6 Table). Likewise, 2 out of 6 in mouse normal heart[81–87], and 2 out of 15 in human normal heart[88–91] were known regulators of cardiac diseases.

### miRNAs and mRNAs form sexually biased networks in ICM

**Fig 4:**
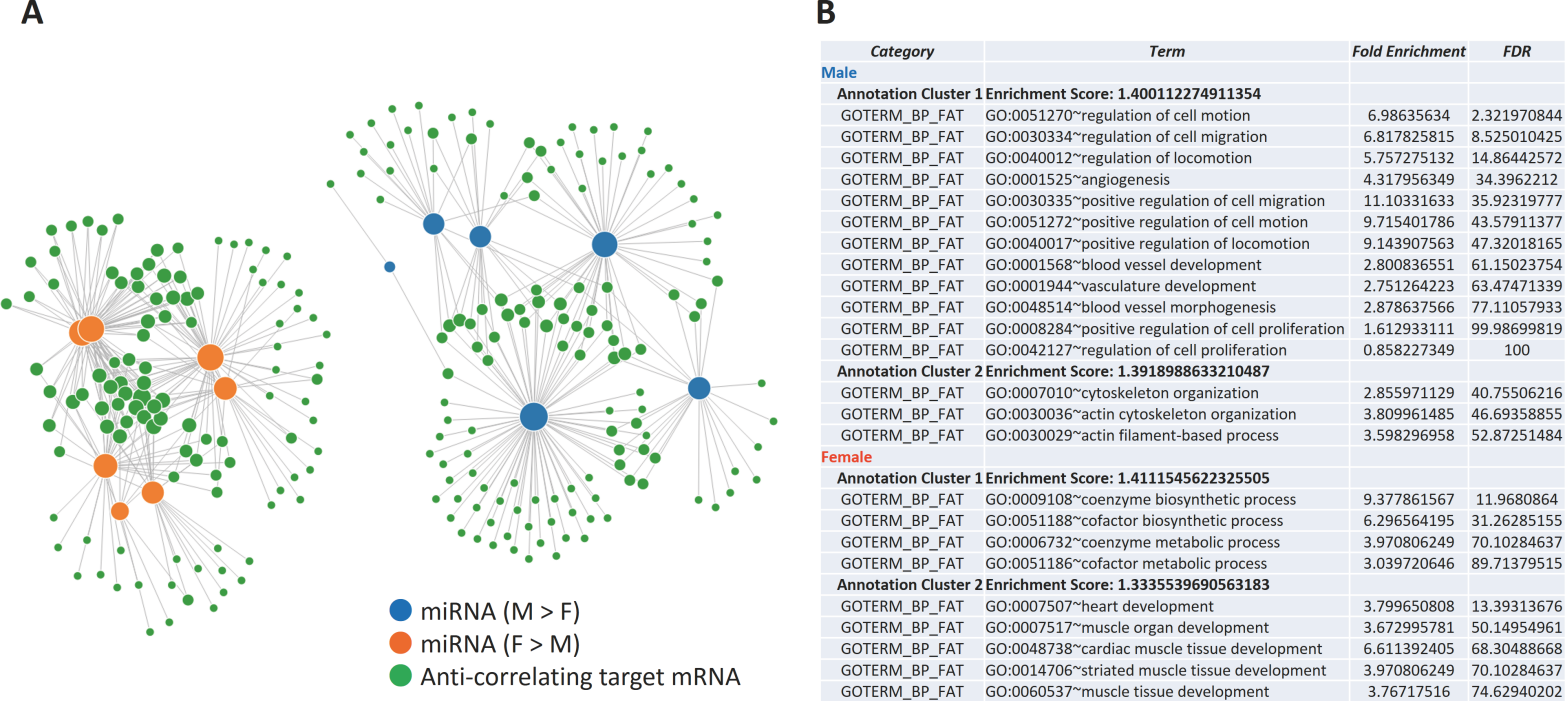
Sexually biased miRNA-mRNA networks in mouse MI. (A) sexually dimorphic miRNAs and mRNAs form sexually biased networks. Blue and orange nodes represent miRNAs expressed higher in male and female, respectively. Green nodes represent target mRNAs. Each link represents one miRNA targeting one mRNA. Node size reflects the number of links. The link length reflects the confidence of the miRNA-mRNA target prediction (the closer, the more confident). A number of genes are targeted by multiple miRNAs, forming sexually biased miRNA-mRNA networks. (B,C) Over-represented GOs of the network component genes specifically down-regulated in male/ female ICM (B). An enrichment score of < 1.3 corresponds to a p-value of < 0.05 (detaled explanation is available as supporting information). See S5 Table for complete list of miRNAs and mRNAs involved.

Since we successfully detected sexually dimorphic mRNAs and miRNAs in ICM, we next searched for sexually dimorphic miRNA-target mRNA relationships from the lists of sexually dimorphic mRNAs, miRNAs, and their predicted targets (S7 Table) in mouse MI. We found that these mRNAs are targeted by multiple miRNAs, forming sexually biased miRNA—mRNA networks (Fig 4A, S7 Table). The genes involved in the male-biased network (hence downregulated in male) over-represented GOs such as angiogenesis, and female-biased network over-represented GOs such as heart development (Fig 4B).

## Discussion

In this work, by integrating the existing mRNA microarray data of mouse and human, we found a number of sexually dimorphic genes in normal heart, ICM, HCM, and DCM, confirming the previous literature and further revealing new genes. These genes over-represented GOs important for heart homeostasis. We further asked if similar sex difference also exists in miRNA transcriptome, and by conducting miRNA microarray analysis of murine MI models and human ICM patients, we found that the miRNA transcriptome shows significant sex difference. Many of these miRNAs were known regulators of cardiac diseases. Computational analysis revealed that these sexually dimorphic miRNAs likely form sexually biased miRNA-mRNA networks in ICM, which potentially impact the prognosis. This offers the first comprehensive picture of the sex difference in mammalian cardiac diseases (ICM, HCM, DCM) at the level of mRNA transcriptome, and for the first time reports the sex difference of diseased heart at the level of miRNA.

### Basal/ disease heart shows sex difference at the level of mRNA transcriptome

Thus far mRNA-level sex difference in mouse MI[44], HCM[31,32,45], DCM[39], and human DCM[46,47] have been reported separately. However, such data of human ICM and HCM are currently lacking. Importantly, no report has provided the picture of transcriptome-level sex differences in heart disease in a comprehensive fashion. Thus, in this study we set out to comprehensively characterize the genetic program underlying the heart sexual dimorphisms.

Meta-analysis is becoming increasingly powerful as the registered expression microarray data accumulate in the GEO database. Here we adopted this technique to investigate the transcriptome-wide sex difference of the heart. The result showed that each cardiac disease has a number of sexually dimorphic genes. The numbers of sexually dimorphic genes in diseased murine heart detected in this meta-analysis were roughly comparable to those reported in at least 2 of the 4 original studies, although the other 2 studies could not be compared to our results because they reported the sex difference without discriminating the healthy and disease samples. By analyzing the raw data of each study separately, we confirmed that an unexpectedly small number of sexually biased genes in normal murine heart (< 100) is attributable to the little overlap of the results of the original studies, and that our result reflects the overlapped genes (data not shown). These observations support the validity of our meta-analysis results.

Interestingly, our results suggest that some of the sexually dimorphic genes are common to some or all of the 4 diseases (Fig 2C and 2D). Since women have less occurrence but have higher risk after establishment of ICM[3–13] and HCM[14–18], sexually dimorphic genes common to ICM and HCM are potentially good targets for gender-specific treatments. Likewise, sexually dimorphic genes in DCM might be good candidates of drug targets since men are protected from DCM[19–25].

An important limitation of the human part of our study is the intergroup differences. Analysis of human disease transcriptome is often complicated due to the confounding biological noises such as age, body habitus, race, comorbidities, and medications. Although we could control for intergroup age differences (S13 Fig), the nature of our analysis—the integration of transcriptome data conducted in different labs with different aims—made it hard to control for the other variables. It is possible that these intergroup differences are underlying relatively small number of sexually biased genes detected in human, especially for disease samples where the sample sizes of women were relatively limited (Fig 2). Still, the set of sexually dimorphic genes presented in this study offers the first valuable clue to understanding the genetic program underlying the sexual dimorphism in heart disease. As for mouse, our analysis did not suffer from this problem because each of these data was generated to identify sexually dimorphic genes. Still, it is important to note that one of these data (Sham + DCM) was generated from a genetic background (BALB6/cJ) different from the rest (C57BL/6). This calls for a caution in directly comparing our data of DCM with the other health conditions. Concerning the validity of the sex difference of each health condition, on the other hand, this DCM data of BALB6/cJ was the only data used to compute the sex difference in DCM. This means that, in exploring the sex difference of heart diseases, samples of different genetic backgrounds are handled practically separately. Hence, the heterogeneity in the genetic background is unlikely to confound our result in this respect.

### Normal/ ICM heart shows sex difference at the level of miRNA transcriptome

mRNAs have been thought to be the primary players in genetic programs. Accordingly, researchers studying the sex difference of mouse/ human cardiac diseases have focused on the mRNA transcriptome[31,32,39,44–47]. However, evidence is accumulating that many aspects of cardiac diseases are critically influenced by miRNAs[48]. Indeed, sexually biased miRNAs have been implicated in sexually dimorphic diseases such as neurodegenerative disorders[92] and metabolic syndrome[93]. It is therefore natural to hypothesize their role in the sex difference of cardiac disease. Consistently, we discovered several miRNAs in disease heart differentially expressed between sexes (Fig 3, Table 3).

The list of sexually dimorphic miRNAs presented in this study, however, is likely only a tip of the iceberg—the fact that male and female can be accurately discriminated on the basis of their transcriptomic PCs indicates that a larger number of genes are differentially expressed between sexes (Fig 3A, 3B, 3E and 3F). As for our mouse data, one factor that might have limited the detection power is the effect of estrous cycle. We did not match the estrus cycle of females in hope of capturing the “average” sexual dimorphism. This likely resulted in the hyper variability of the female samples. In support of this, correlation between female samples were significantly weaker than males in MI (S14 Fig), although in sham, the sample sizes were too small to assess the correlation difference. Consistently, larger number of genes showed > 1.2 fold sex differences (99 and 23 genes in sham and MI, respectively) than genes deemed statistically significant (6 and 13 genes in sham and MI, respectively). We predict that a population of miRNAs larger than detected in this study are differentially expressed between sexes. Additional studies on larger number of samples will be of considerable interest.

### Sexually dimorphic miRNAs might be regulated by sex hormones

The expression of sex-biased miRNAs could stem from both sex chromosome and sex hormone effects[94]. X-chromosome is highly enriched in miRNAs, and approximately 15% of genes encoded by the inactive X-chromosome in humans escape inactivation[95], although in mouse this extent appears less[96]. We thus checked the chromosomal enrichment of the sexually dimorphic miRNAs in disease heart but found no such enrichment (S12 Fig). Sex steroid hormones–estradiol, progesterone and testosterone–also have been suggested to regulate miRNA expression in the context of cancer and brain[97,98]. Also supporting the role of sex hormones are studies reporting evidence that sex hormones induce the sex difference of genes in heart, albeit conflicting[31–33,36,38,40–43]. It would be interesting to assess this hypothesis, for example by measuring the binding of sex hormone receptors to the promoters of sexually dimorphic miRNAs. Measuring this by ChIP analysis and comparing it between sexes would provide a good insight into this hypothesis. Also, counterintuitive as it may seem that we observed miRNA sex differences in patients of > 70 years of age, literature do exist that report differences in autosomal gene expression in men vs postmenopausal women[99,100]. Although neither of these reports discusses the possible mechanisms of autosomal sex differences, they do support the existence of post-menopausal sex difference in autosomal gene expression.

### Sexually biased miRNA-mRNA networks operate in ICM

We found that in murine ICM model, sexually dimorphic miRNAs seem to target the sexually dimorphic genes, forming sexually biased miRNA-mRNA networks. The genes repressed in male and female over-represented GOs such as angiogenesis and heart development, respectively (Fig 4B). This supports the following scenario: after developing ICM, male heart represses genes involved in angiogenesis, leading to the worse prognosis. In female heart, genes involved in heart development are repressed, preventing the harmful reactivation of the fetal cardiac gene program[101]. Although this network analysis was not applicable to human ICM due to the limited number of sexually dimorphic miRNAs detected, considering the shared phenotypic sex differences in cardiac diseases, the sexually biased networks revealed in mouse MI likely operate in human as well.

Note an important limitation that the miRNAs and mRNA data were not obtained from the same samples nor from samples of the same timing post MI (miRNA from chronic MI versus mRNA from acute MI). This means that the miRNA and mRNA data presented in this work are, strictly speaking, not directly comparable. Unfortunately the strong batch effect observed in our samples, which was effectively removed in our microarray data on the basis of transcriptome, did not allow us to directly confirm the sexual dimorphism of these genes in our samples by qPCR. Confirming the sexual dimorphism of these genes using batch-free chronic MI samples would be an important next step.

If the presented networks indeed operate and cause symptomatic sex differences in ICM, simultaneously targeting the components of these networks might enable highly effective and specific treatment strategies. In addition, considering the shared phenotypic sexual dimorphisms of the other cardiac diseases such as HCM and DCM, it is conceivable that sexually biased networks similarly operate in these cardiac diseases. Future miRNA microarray analyses are awaited to clarity this tempting possibility.

In summary, this study comprehensively characterized the sex difference of cardiac diseases at the level of miRNA and mRNA transcriptome, laying the foundation for the gender specific treatment strategies.

## Conclusions

The existing mRNA microarray data of both mouse and human heart were integrated, identifying sexually dimorphic genes in cardiac diseases (ICM, HCM, and DCM). These genes over-represented GOs essential for heart homeostasis. Furthermore, microarray of miRNA isolated from mouse/ human ICM and normal heart samples was conducted, identifying sexually dimorphic miRNAs. Computational analysis revealed miRNA-mRNA networks that operate in a sexually biased fashion. Together this study provides the first comprehensive picture of the genome-wide program underlying the heart sexual dimorphisms, laying the foundation for the gender specific treatment strategies.

## Ethics approval and consent to participate

Human tissue samples, acquired during post-mortem examination and frozen in liquid nitrogen, were provided by the department of pathology, Tokyo Metropolitan Geriatric Hospital after the approval from the ethical committee.

Our experimental procedures and protocols of animals were approved by the Committee for Animal Research, Kyoto Prefectural University of Medicine, and performed in accordance with the US Animal Welfare Act.

## Supporting information

S1 Fig PCA of the mRNA microarray metadata before/ after batch effect correction. (A-D) PCA of (A, B) mouse and (C, D) human metadata before batch effect correction. (E-F) PCA of (E, F) mouse and (G, H) human metadata after batch effect correction. Each dot represents each sample, colored by (A, E, C, G) batch or (B, F, D, H) disease. Samples of the same health condition are clustered together after normaliztion, indicating that normalization effectively removed the batch effect.
(TIF)

S2 Fig Sex discrimination in the mRNA microarray metadata by LDA based on the principal components. Sex was clearly discriminated in each disease. (A-D) mouse, (E-H), human. (A,E) Normal, (B,F) ICM, (C,G) HCM, and (D,H) DCM. The LOOCV error rate and its p-value are shown in each graph. Shown curves are the fitted normal distributions of each sex.
(TIF)

S3 Fig The cumulative variance explained by the PCs used for the discriminant analysis in S2 Fig. (A-D) mouse, (E-H), human. (A,E) Normal, (B,F) ICM, (C,G) HCM, and (D,H) DCM.
(TIF)

S4 Fig **The weight distribution of PC1 used for the discriminant analysis in S2 Fig** (A-D) mouse, (E-H), human. (A,E) Normal, (B,F) ICM, (C,G) HCM, and (D,H) DCM. PC weights are not dominated by small number of genes, but rather it appears many genes contribute to PC1. The other PCs showed similar weight distributions (data not shown).
(TIF)

S5 Fig LDA of diseases in the mRNA microarray metadata. (A-C) mouse, (D-F) human. (A,D)LDA results. The LOOCV error rate and its p-value are shown above each plot. (B,E) the cumulative variance explained by PCs used for LDA. (C,F) The weight distribution of PC1.
(TIF)

S6 Fig **Chromosome enrichment of sexually dimorphic mRNAs of (A,B,E,F) mouse, (C,D,G,H) human**. (A,C) male-biased genes in normal heart, (B,D) female-biased genes in normal heart. (E,G) male-biased genes in ICM, (F,H) female-biased genes in ICM. Light blue: the ratio of chromosome of the biased genes detected in this study. Dark blue: the ratio of chromosome of all the genes considered in this study. Sexually biased genes are enriched on several chromosomes when compared with all the genes considered in this study. *p < 0.05.
(TIF)

S7 Fig Mouse chronic MI model samples. Shown are the ventricle side. M: male, F: female, sh: sham, MI: myocardial infarction (e.g. F_MI: female MI). The hearts exhibit post-myocardial infarction left ventricle (LV) remodeling with LV free wall thinning and dilatation. *if the fibrotic change was clearer from dorsal view, dorsal side is shown (labeled *).
(TIF)

S8 Fig qPCR validation of the miRNA microarray platform. X axes show the Cq values of each sample subtracted from that of a reference gene (mmu-miR-23a-3p). Y axes show the signal intensities of the microarray. Both axes are in log2 scale. Overall the miRNA microarray results seem to be consistent with that of qPCR. miRNAs that showed high variance between samples in the microarray show strong consistency between microarray and qPCR (mmu-miR-208b-3p, mmu-miR-3473a, mmu-miR-709). 5 out of 7 randomly chosen miRNAs with significant sex difference post MI also showed significant consistency.
(TIF)

S9 Fig **PC weights of the miRNAs of (A, B) mouse and (C, D) human miRNA array data used for discriminant analysis in Fig 3**. (A, C) normal (B, D) ICM.
(TIF)

S10 Fig The cumulative variance explained by PCs used for discriminant analysis in Fig 3. (A,B) correspond to Fig 3A, 3B, 3C and 3D to Fig 3E and 3F.
(TIF)

S11 Fig Discrimination of healthy and ICM samples by LDA based on the principal components of the miRNA array data. Normal and ICM samples are well discriminated. (A) mouse (B) human. Red indicates ICM and blue indicates normal heart. The LOOCV error rate is shown in each graph. Shown curves are the fitted normal distributions of each health condition. The LOOCV error rate is shown in each graph.
(TIF)

S12 Fig **Chromosome enrichment of sexually dimorphic miRNAs of (A,B,E,F) mouse and (C,D,G,H) human**. (A,C) male-biased genes in normal heart, (B,D) female-biased genes in normal heart. (E,G) male-biased genes in ICM, (F,H) female-biased genes in ICM. Light blue: the ratio of chromosome of the biased genes detected in this study. Dark blue: the ratio of chromosome of all the genes considered in this study. Sexually biased genes are enriched on several chromosomes when compared with all the genes considered in this study. *p < 0.05.
(TIF)

S13 Fig Age of the patients used in the meta-analysis. Within each disease, age was effectively controlled between genders. NR: normal. * p < 0.05, n.s. not significant.
(TIF)

S14 Fig Within-group correlation plot of mouse miRNA microarray data. Correlations between all possible pairs of samples of the same group (e.g. female ICM) are plotted. (A) sham (B) ICM. In ICM, female samples have lower correlations than male. Welch’s two-sample t-test was used. *p < 0.05.
(TIF)

S1 Table Sexually dimorphic mRNAs of mouse identified by microarray meta-analysis. (XLSX)

S2 Table Sexually dimorphic mRNAs of human identified by microarray meta-analysis. (XLSX)

S3 Table Over-represented GO clusters of the sexually dimorphic mRNAs in mouse and human. In mouse NF and human ICM, the number of sexually dimorphic genes detected was not sufficient for clustering analysis and thus the corresponding columns are left blank. Clusters are shown in a decreasing order according to enrichment score (indicated in bracket). Enrichment score > 1.0 (equivalent to p < 0.10) was deemed significant.
(XLSX)

S4 Table miRNAs differentially expressed between sham and MI identified by microarray. (XLSX)

S5 Table GO/ Pathway enrichment analysis of the sexually dimorphic miRNA targets. All clusters presented show enrichment score > 1.3 (equivalent to p-value < 0.05), although individual categories within clusters often show p-values > 0.05.
(XLSX)

S6 Table List of literature reporting the functions of sexually biased miRNAs relevant to cardiac diseases. (XLSX)

S7 Table List of miRNA-gene links comprising the sex-specific networks. In miRupdown’ and ‘mRNAupdown’, 1 means stronger expression in male and -1 in female. miTGscore indicates the confidence of miRNA-mRNA targeting prediction according to the DIANA microT CDS algorhithm.
(XLSX)

S1 Methods Supporting methods. (RTF)
