# Correction: Sexual dimorphisms of mRNA and miRNA in human/murine heart disease

**DOI:** 10.1371/journal.pone.0229750

**Published:** 2020-02-21

**Authors:** Masato Tsuji, Takanori Kawasaki, Takeru Matsuda, Tomio Arai, Satoshi Gojo, Jun K. Takeuchi

An additional affiliation is incorrectly listed for the first author. Masato Tsuji is not affiliated with #1 but only with #2: Department of Biological Sciences, Graduate School of Science, The University of Tokyo, Tokyo, Japan.
