# Supplementary figures and images for: Sexual dimorphisms of mRNA and miRNA in human/murine heart disease
Source: PLoS One. 2017 Jul 13;12(7):e0177988. doi: 10.1371/journal.pone.0177988 (PMC5509429; doi:10.1371/journal.pone.0177988)

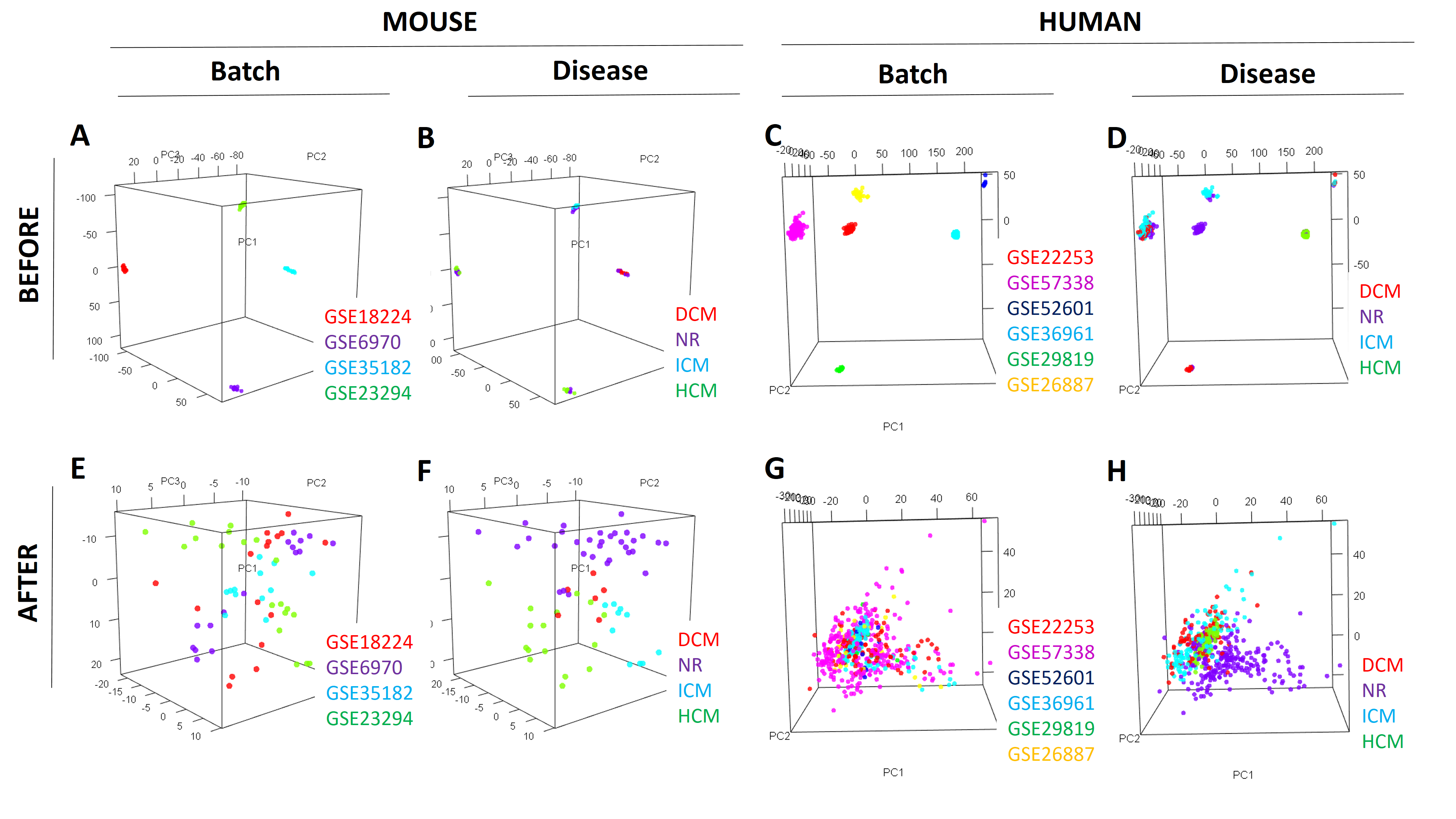

Supplement: S1 Fig — (A-D) PCA of (A, B) mouse and (C, D) human metadata before batch effect correction. (E-F) PCA of (E, F) mouse and (G, H) human metadata after batch effect correction. Each dot represents each sample, colored by (A, E, C, G) batch or (B, F, D, H) disease. Samples of the same health condition are clustered together after normaliztion, indicating that normalization effectively removed the batch effect. (TIF) [file pone.0177988.s001.TIF]

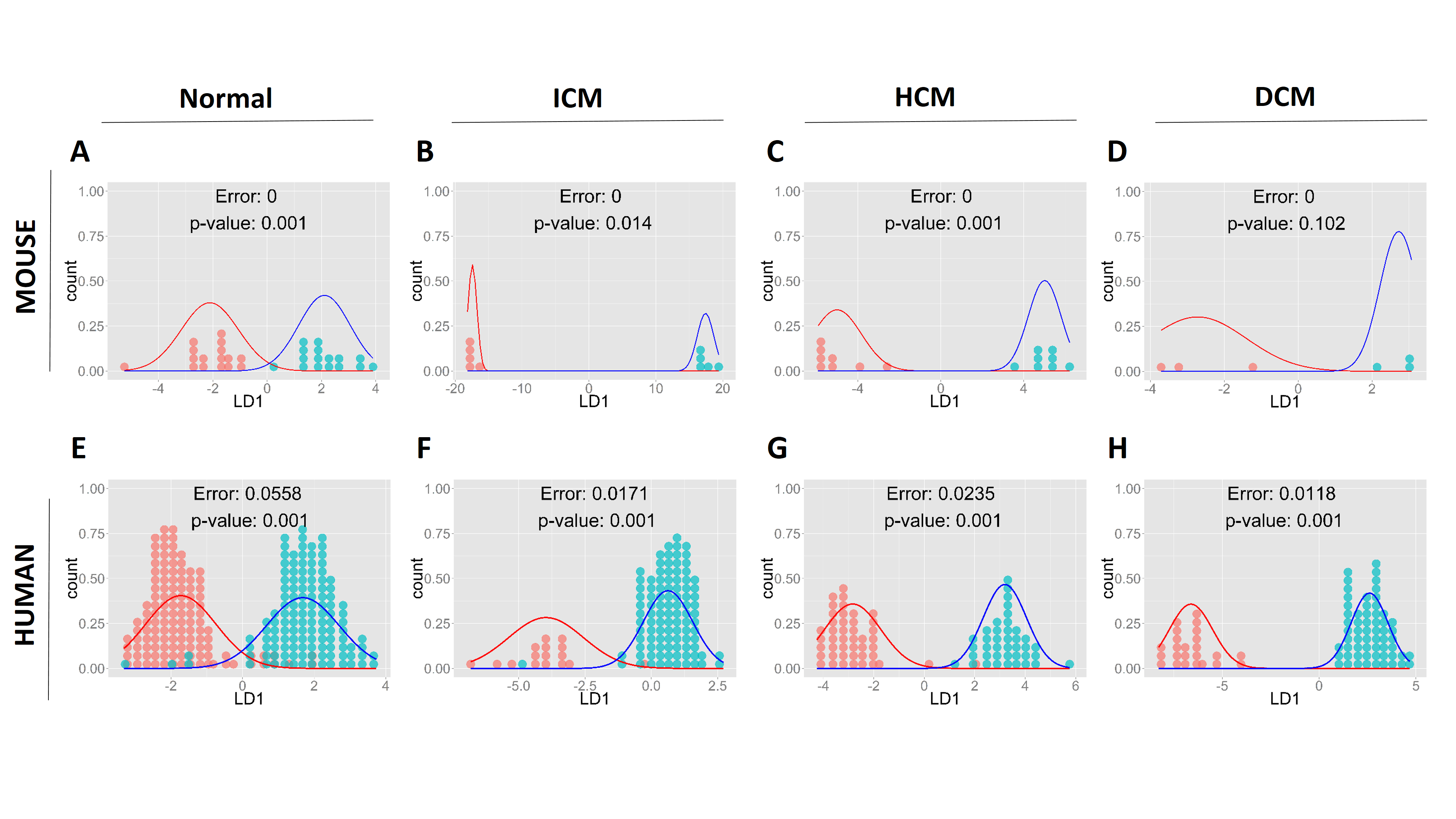

Supplement: S2 Fig — Sex was clearly discriminated in each disease. (A-D) mouse, (E-H), human. (A,E) Normal, (B,F) ICM, (C,G) HCM, and (D,H) DCM. The LOOCV error rate and its p-value are shown in each graph. Shown curves are the fitted normal distributions of each sex. (TIF) [file pone.0177988.s002.TIF]

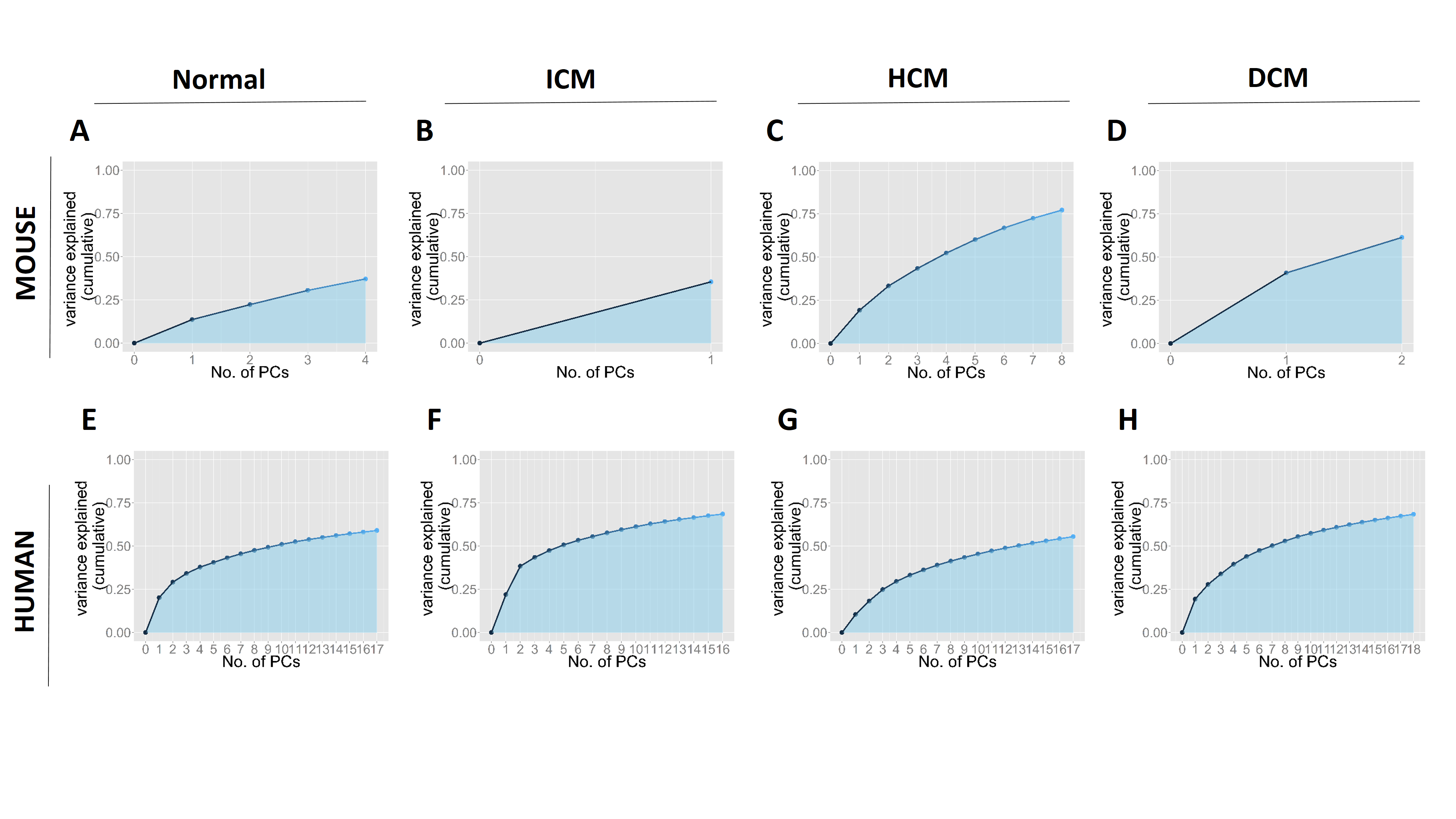

Supplement: S3 Fig — (A-D) mouse, (E-H), human. (A,E) Normal, (B,F) ICM, (C,G) HCM, and (D,H) DCM. (TIF) [file pone.0177988.s003.TIF]

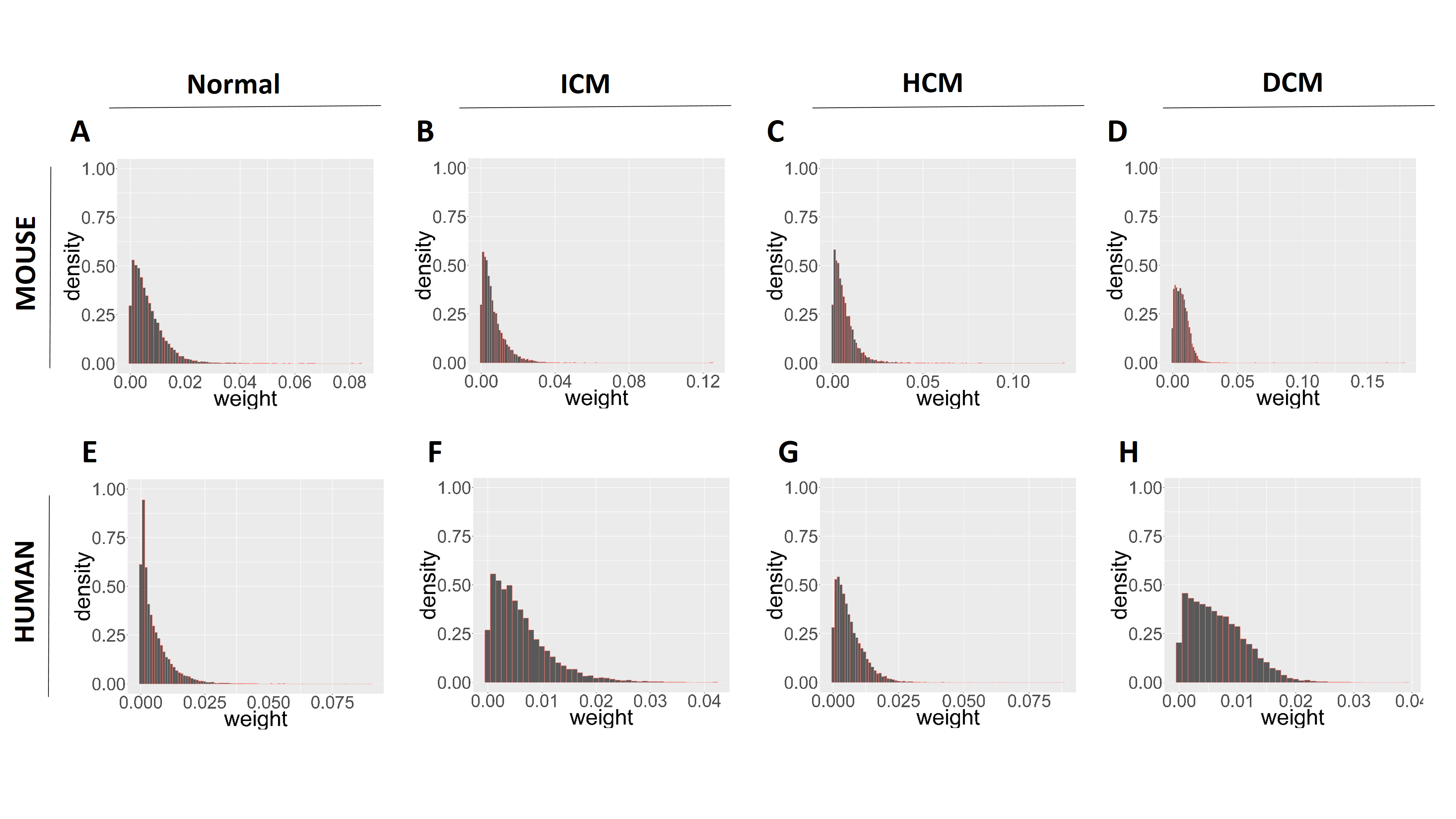

Supplement: S4 Fig — The weight distribution of PC1 used for the discriminant analysis in S2 Fig (A-D) mouse, (E-H), human. (A,E) Normal, (B,F) ICM, (C,G) HCM, and (D,H) DCM. PC weights are not dominated by small number of genes, but rather it appears many genes contribute to PC1. The other PCs showed similar weight distributions (data not shown). (TIF) [file pone.0177988.s004.TIF]

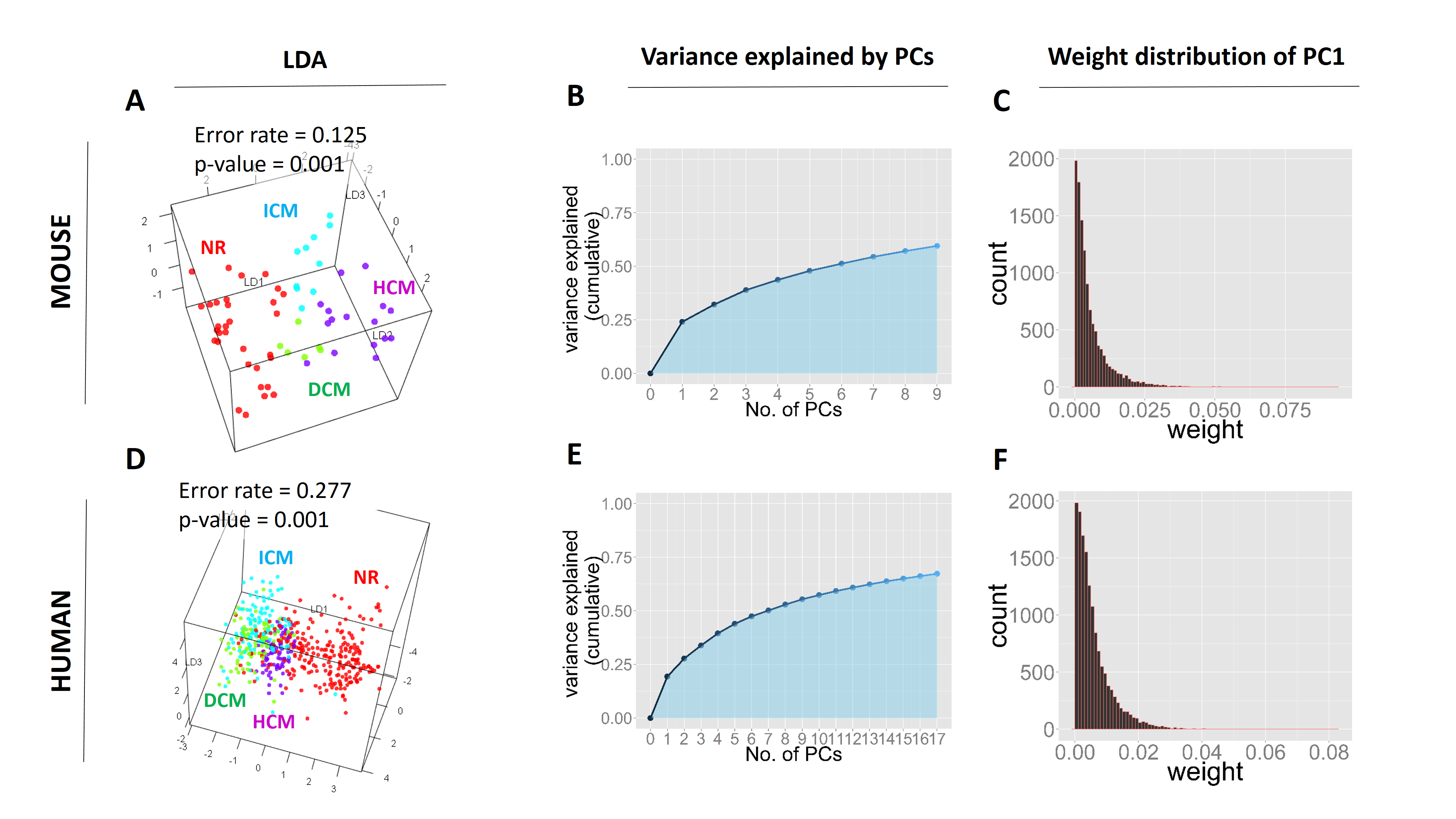

Supplement: S5 Fig — (A-C) mouse, (D-F) human. (A,D)LDA results. The LOOCV error rate and its p-value are shown above each plot. (B,E) the cumulative variance explained by PCs used for LDA. (C,F) The weight distribution of PC1. (TIF) [file pone.0177988.s005.TIF]

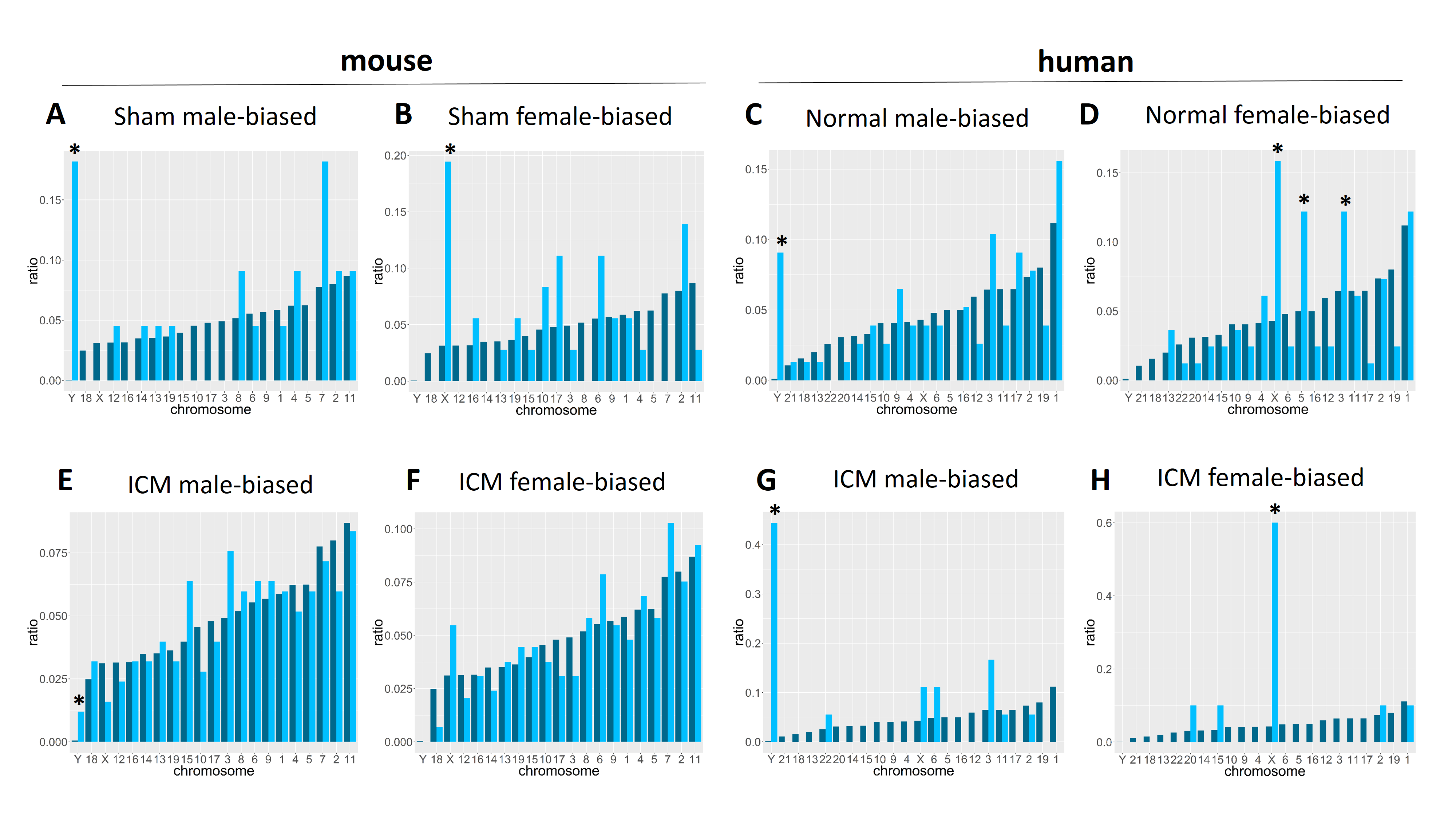

Supplement: S6 Fig — Chromosome enrichment of sexually dimorphic mRNAs of (A,B,E,F) mouse, (C,D,G,H) human. (A,C) male-biased genes in normal heart, (B,D) female-biased genes in normal heart. (E,G) male-biased genes in ICM, (F,H) female-biased genes in ICM. Light blue: the ratio of chromosome of the biased genes detected in this study. Dark blue: the ratio of chromosome of all the genes considered in this study. Sexually biased genes are enriched on several chromosomes when compared with all the genes considered in this study. *p < 0.05. (TIF) [file pone.0177988.s006.tif]

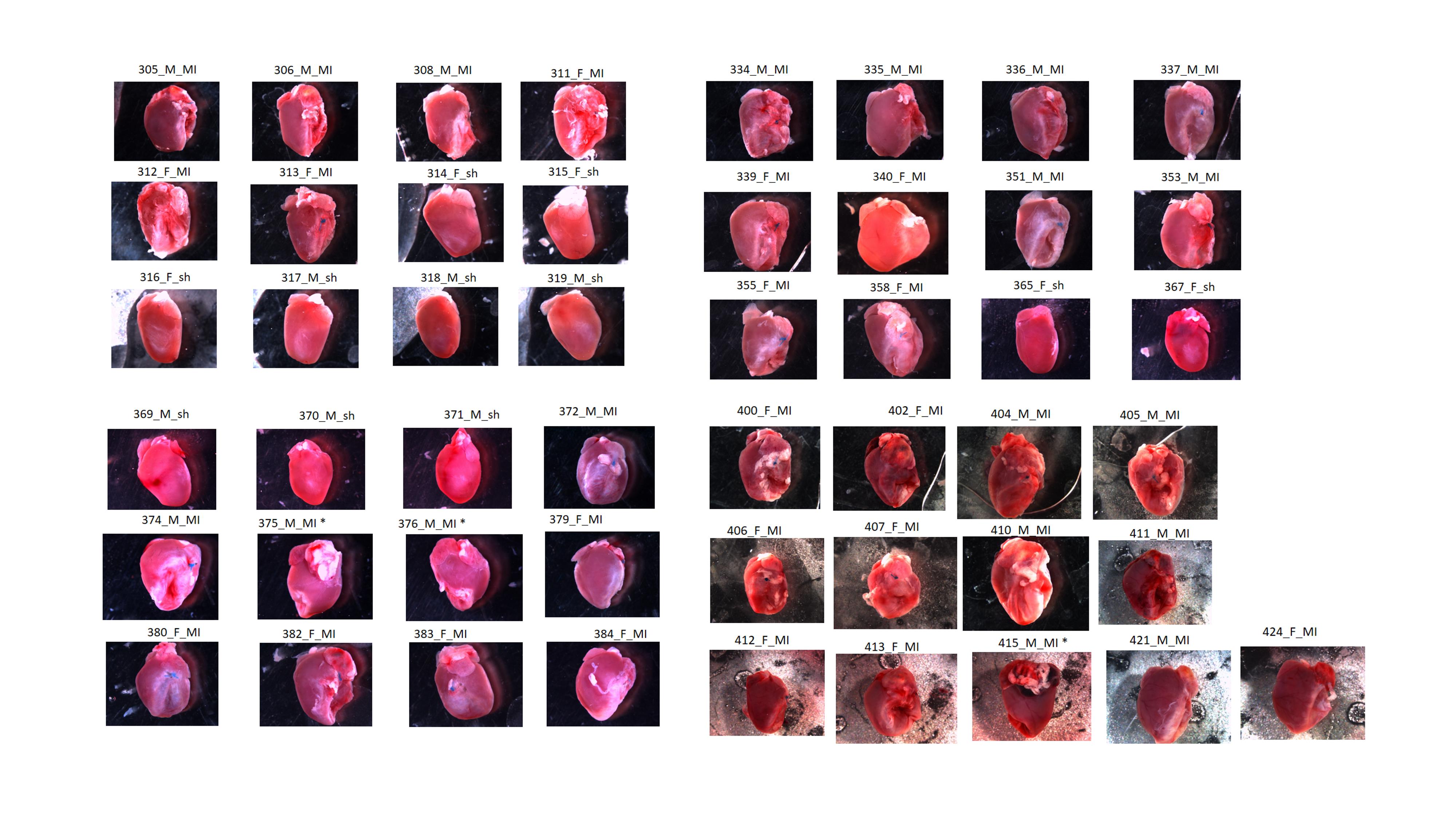

Supplement: S7 Fig — Shown are the ventricle side. M: male, F: female, sh: sham, MI: myocardial infarction (e.g. F_MI: female MI). The hearts exhibit post-myocardial infarction left ventricle (LV) remodeling with LV free wall thinning and dilatation. *if the fibrotic change was clearer from dorsal view, dorsal side is shown (labeled *). (TIF) [file pone.0177988.s007.tif]

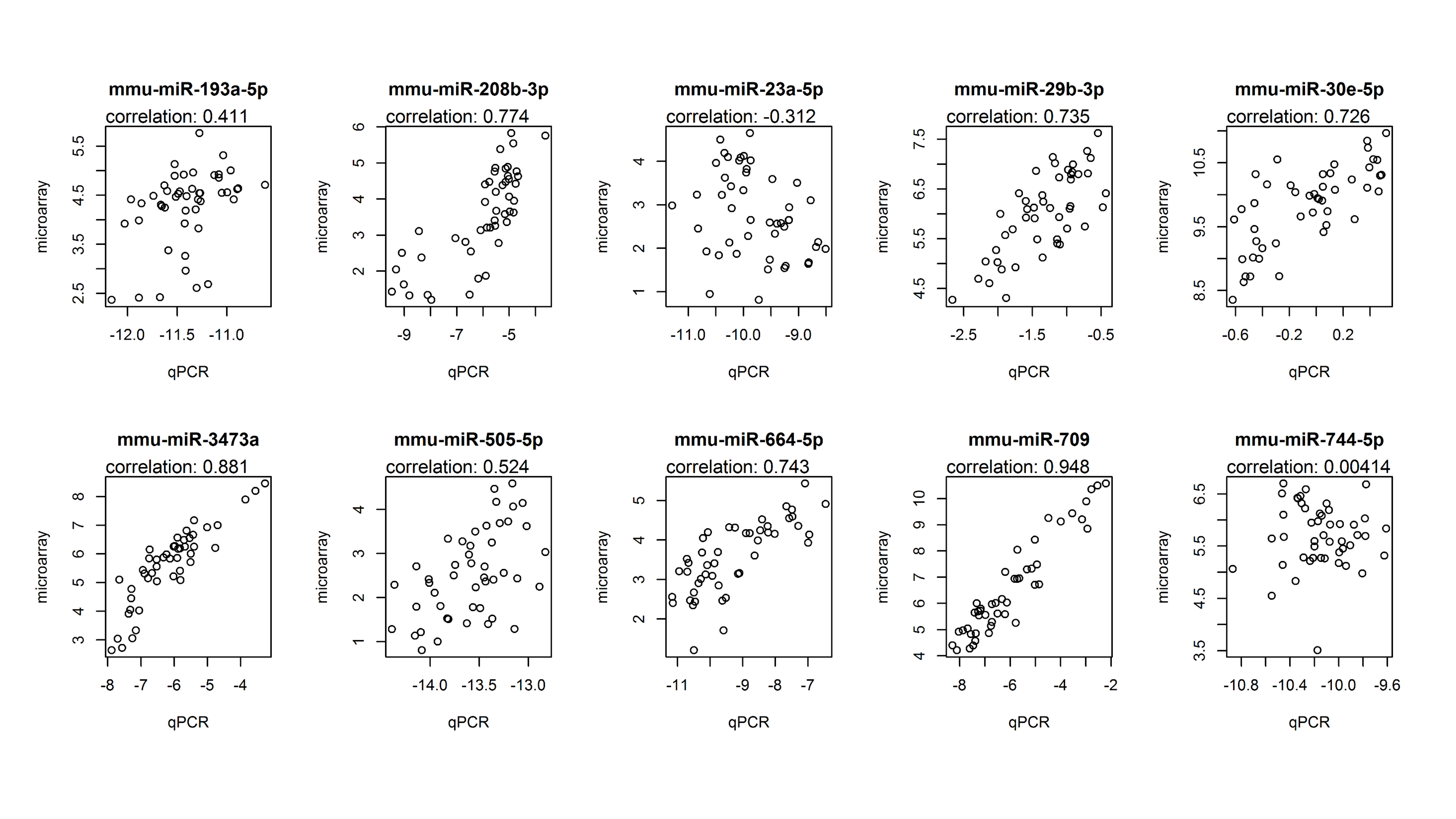

Supplement: S8 Fig — X axes show the Cq values of each sample subtracted from that of a reference gene (mmu-miR-23a-3p). Y axes show the signal intensities of the microarray. Both axes are in log2 scale. Overall the miRNA microarray results seem to be consistent with that of qPCR. miRNAs that showed high variance between samples in the microarray show strong consistency between microarray and qPCR (mmu-miR-208b-3p, mmu-miR-3473a, mmu-miR-709). 5 out of 7 randomly chosen miRNAs with significant sex difference post MI also showed significant consistency. (TIF) [file pone.0177988.s008.TIF]

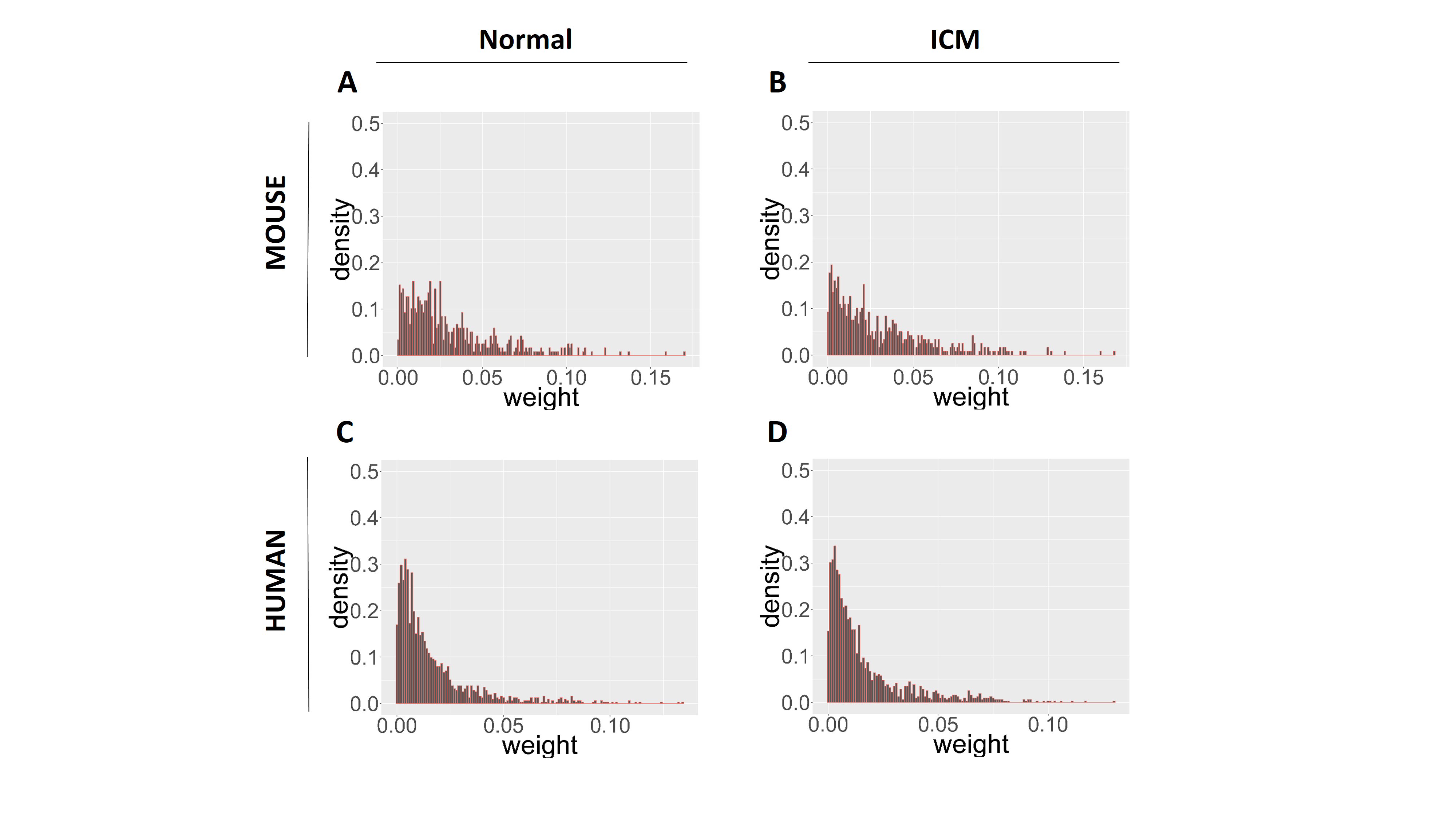

Supplement: S9 Fig — PC weights of the miRNAs of (A, B) mouse and (C, D) human miRNA array data used for discriminant analysis in Fig 3. (A, C) normal (B, D) ICM. (TIF) [file pone.0177988.s009.TIF]

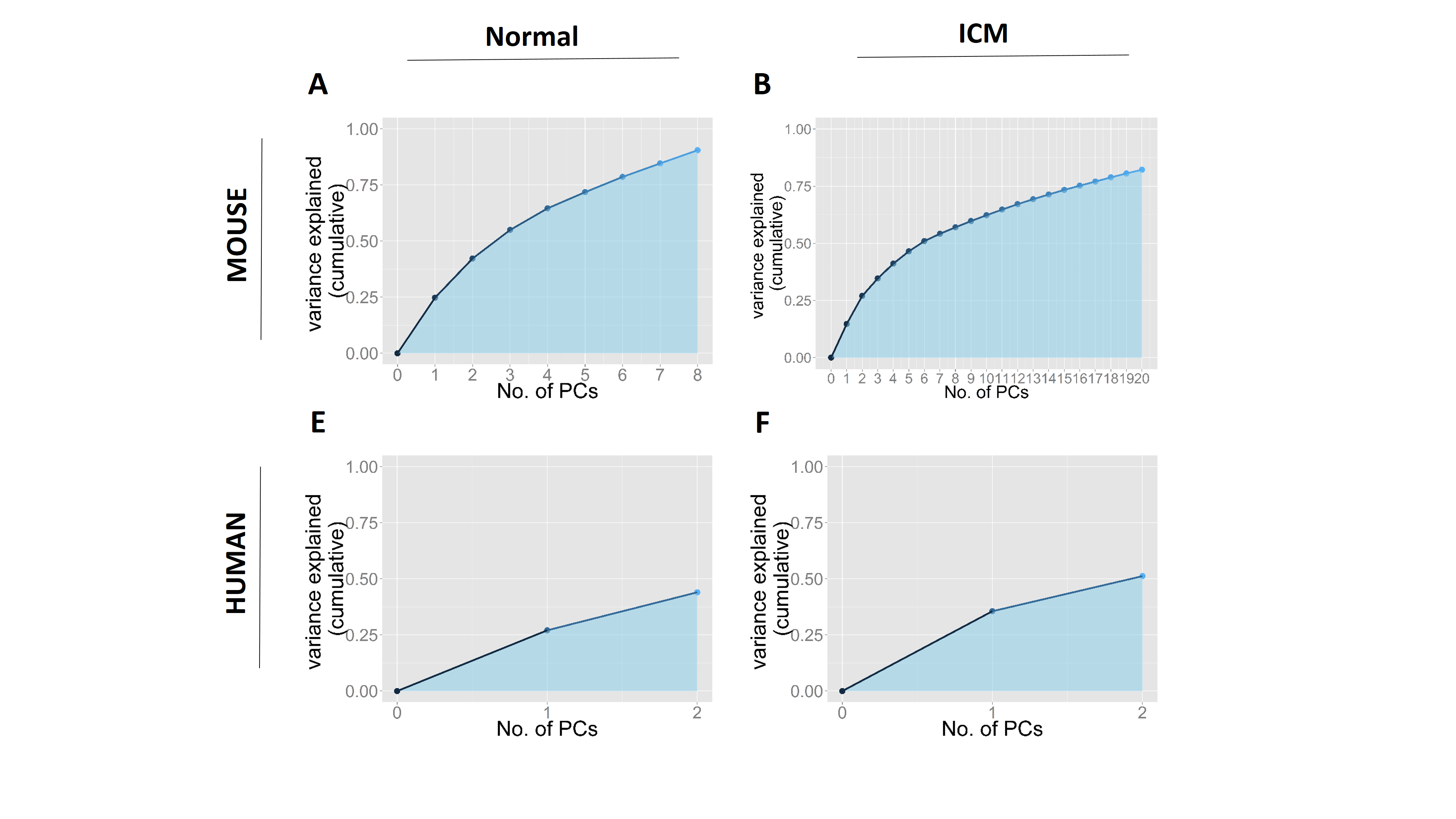

Supplement: S10 Fig — (A,B) correspond to Fig 3A, 3B, 3C and 3D to Fig 3E and 3F. (TIF) [file pone.0177988.s010.TIF]

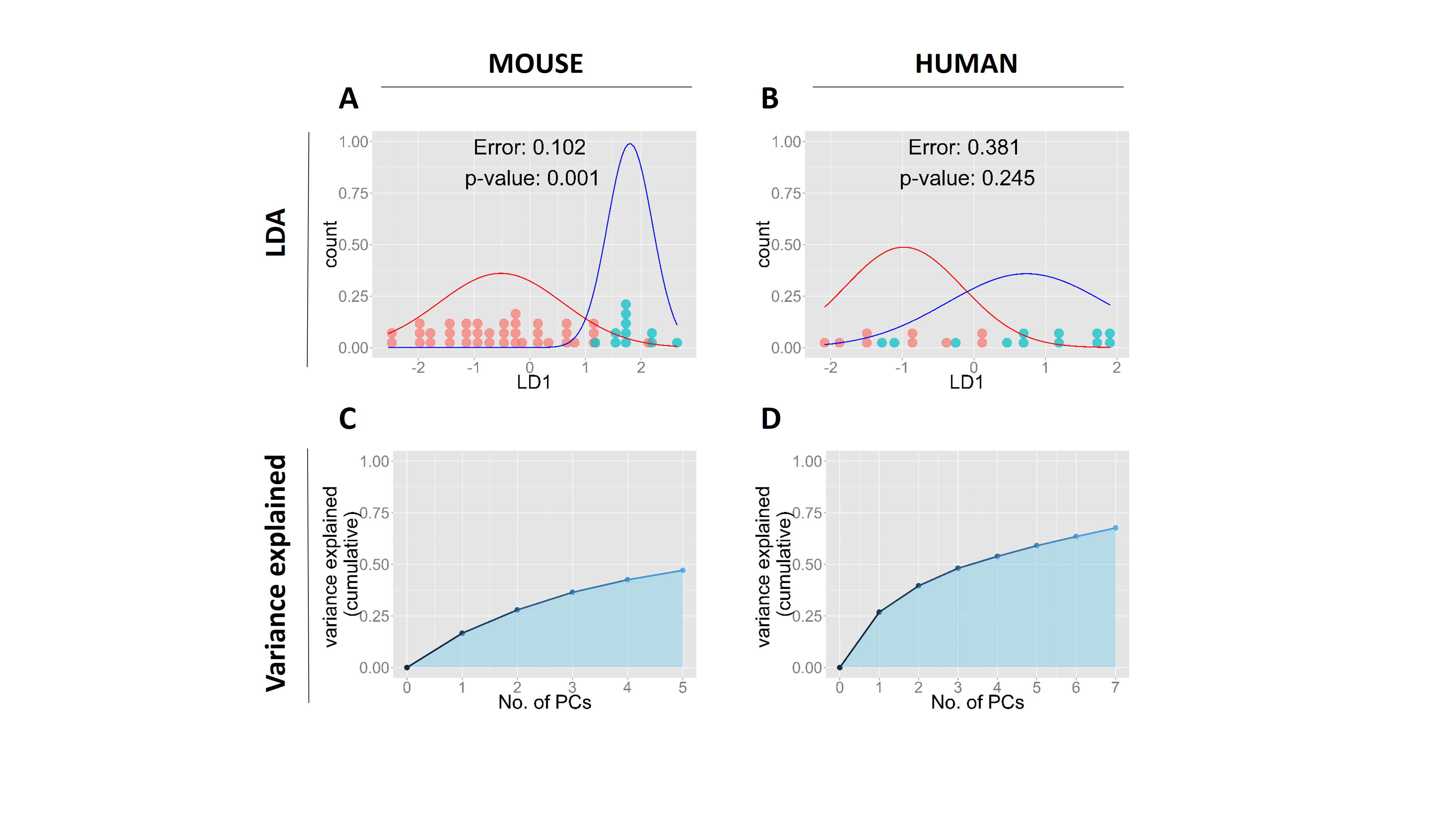

Supplement: S11 Fig — Normal and ICM samples are well discriminated. (A) mouse (B) human. Red indicates ICM and blue indicates normal heart. The LOOCV error rate is shown in each graph. Shown curves are the fitted normal distributions of each health condition. The LOOCV error rate is shown in each graph. (TIF) [file pone.0177988.s011.TIF]

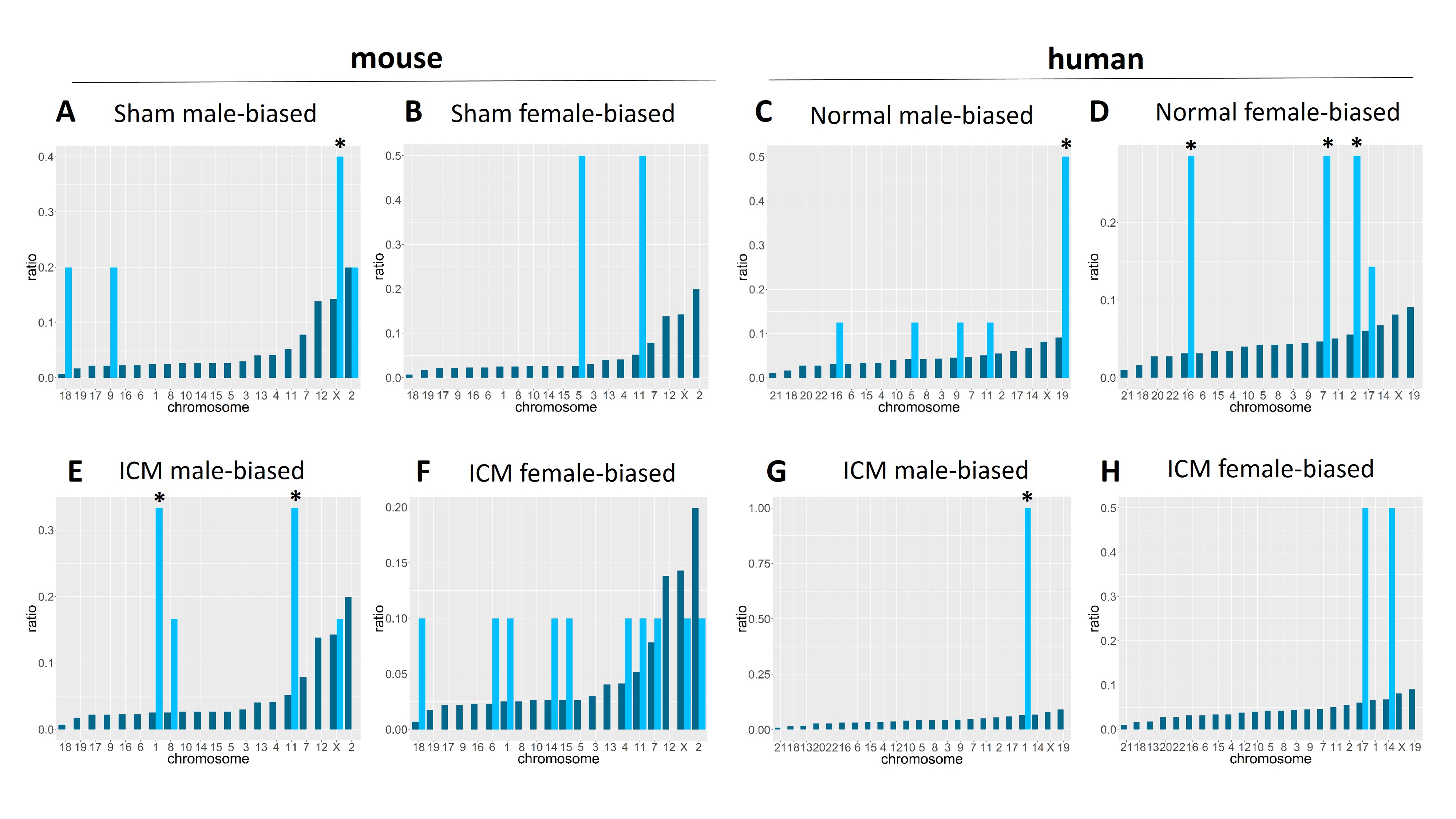

Supplement: S12 Fig — Chromosome enrichment of sexually dimorphic miRNAs of (A,B,E,F) mouse and (C,D,G,H) human. (A,C) male-biased genes in normal heart, (B,D) female-biased genes in normal heart. (E,G) male-biased genes in ICM, (F,H) female-biased genes in ICM. Light blue: the ratio of chromosome of the biased genes detected in this study. Dark blue: the ratio of chromosome of all the genes considered in this study. Sexually biased genes are enriched on several chromosomes when compared with all the genes considered in this study. *p < 0.05. (TIF) [file pone.0177988.s012.tif]

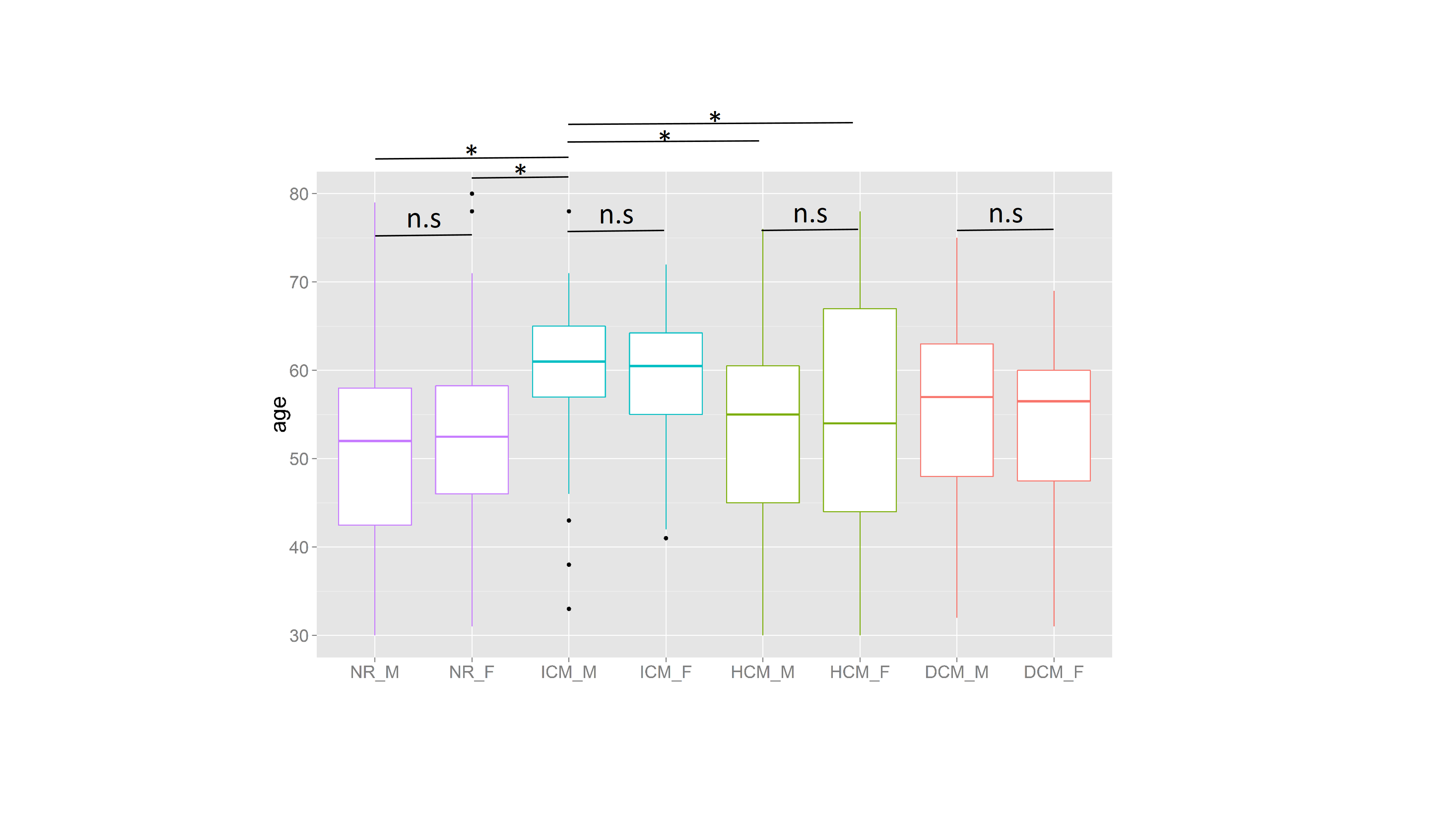

Supplement: S13 Fig — Within each disease, age was effectively controlled between genders. NR: normal. * p < 0.05, n.s. not significant. (TIF) [file pone.0177988.s013.TIF]

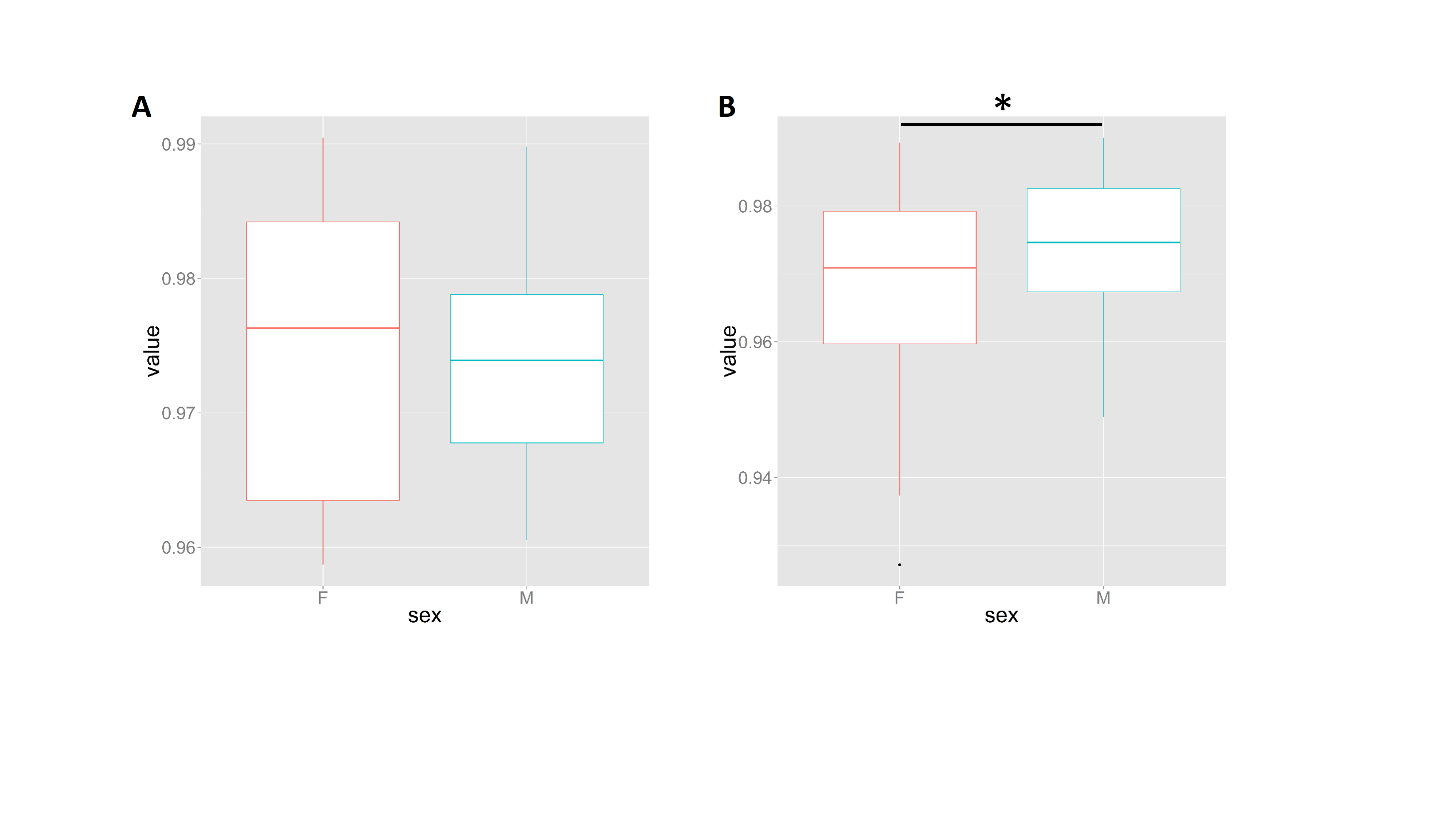

Supplement: S14 Fig — Correlations between all possible pairs of samples of the same group (e.g. female ICM) are plotted. (A) sham (B) ICM. In ICM, female samples have lower correlations than male. Welch’s two-sample t-test was used. *p < 0.05. (TIF) [file pone.0177988.s014.TIF]
