# Supplementary material for: Sexual dimorphisms of mRNA and miRNA in human/murine heart disease
Source: PLoS One. 2017 Jul 13;12(7):e0177988. doi: 10.1371/journal.pone.0177988 (PMC5509429; doi:10.1371/journal.pone.0177988)
Supplement: S1 Methods — (RTF) [file pone.0177988.s022.rtf]

MethodsAll microarray data have been submitted to the National Center for Biotechnology Information gene expression and hybridization array data repository (GSE76604). All studies were conducted in accordance with protocols approved by the University of Tokyo Institutional Review Boards.Myocardial infarction modelingExperimental procedures and protocols were approved by the Committee for Animal Research, Kyoto Prefectural University of Medicine, and performed in accordance with the US Animal Welfare Act. Both C57BL/6 male and female mice, aged 10 weeks and weighing 23–28 g, were used. General anesthesia was maintained under a ventilator with isoflurane. Following left thoracotomy, 8-0 Prolene™ suture material was passed beneath the left anterior descending coronary artery just distal to the main trunk, and a knot was tied at that level to create extensive myocardial infarction. The sham surgeries were performed by pericardiotomy through left thoracotomy. There was no significant difference in the post-operative mortality between sexes during this period (data not shown). Before sampling the myocardial tissues, the hearts were macroscopically validated to exhibit post-myocardial infarction left ventricle (LV) remodeling with LV free wall thinning and dilatation (S7 Fig). The ventricular septum of the areas at risk of ischemia was sampled on post-operative day 28 to examine the gene profiles. Patient selection and tissue collectionHuman tissue samples were provided by the department of pathology, Tokyo Metropolitan Geriatric Hospital after receiving informed written consent for this study by he families (the bereaved) under the Act of Post-mortem examination and the approval from the Ethics Committe. Age- and sex-matched cohorts were selected to compare healthy hearts to those with post-MI LV remodeling. Among the patients’ cardiac demographics, patients suffering from ventricular hypertrophy and any valvular disorders were excluded to reduce the biological complexities. We focused on the septuagenarian and octogenarian populations that were most liable to be affected by post-MI LV remodeling. LV samples, including the area at risk of ischemia (border zone for myocardial infarction), were acquired during post-mortem examination and frozen in liquid nitrogen. RNA extractionOne month after MI of murine model, the border zone of the infarct (or corresponding region of sham samples) was surgically dissected in ice-cold PBS. All samples were thoroughly mashed in ice-cold Sepasol (Sepasol-RNA I super G, nakalai tesque, Japan) with biomasher (BioMasher II, nippi, Japan). Large RNA and small RNA were extracted separately with the NucleoSpin miRNA kit (MACHEREY-NAGEL, Germany). RNA sample concentration and integrity were determined using spectrophotometry (Nanodrop 1000; Thermo Scientific, USA) and a Bioanalyzer (Bioanalyzer 2100; Agilent Technologies, USA) with RNA 6000 Nano Labchip, although Bioanalyzer was alternated for human samples with a manual inspection of the r18S-r28S ratio of the gel electrophoresis results. Only samples with an average RNA integrity number >7 were used.RNA library preparation, microarray, and data processingAfter a brief tailing reaction with polyA, miRNA samples of 250 – 280 ng were labeled by FlashTag ligation biotin mix. Labeled RNAs were hybridized overnight to Affymetrix GeneChip® miRNA 3.0 Arrays containing probes for 19,724 mature miRNAs derived from the Sanger miRBase V17 (from 153 organisms). Each array included probes for 1,111 mouse mature miRNAs and 1,733 human mature miRNAs. After hybridization, arrays were washed and stained according to standard Affymetrix protocol and then scanned on an Affymetrix GeneChip® Scanner 3000. Quality control was conducted using the Affymetrix® Expression Console™ Software and standard Affymetrix quality metrics. Raw microarray data were background corrected, log transformed, and quantile normalized using the robust multi-array average (RMA) algorithm as implemented in Affymetrix® Expression Console™ Software. Since mouse samples were prepared in several steps, the resulting batch effects were removed with COMBAT method[1] as implemented in sva package in R[2]. Public microarray data integrationRelevant studies were searched in the NCBI Gene Expression Omnibus (GEO) and their raw data were downloaded and processed in R according to each array platform, removing the batch effect by COMBAT method[1] if necessary. As for mouse, we used 4 studies (GSE23294[3], GSE18224[4,5], GSE6970[6], GSE35182[7]) that reported the transcriptome of either Sham/ MI/ HCM/ DCM model mouse heart of both sexes with the information of sex for each sample (Table1). Although GSE35182 - reporting the DCM transcriptome induced by CBV3 viral infection - included samples of both 10 days and 90 days post infection (dpi), we used only 90 dpi data to match with the human situation. As for human, we used 6 studies (GSE57338[8], GSE29819[9], GSE22253[10], GSE26887[11], GSE52601[12], GSE36961 (Hebl VB, Bos JM, Oberg AL, Sun Z, Herman DS, Teekakirikul P, Seidman JG, Seidman CE, dos Remedios CG, Schaff HV, Dearani JA, Ommen SR, Brozovich FV, Ackerman MJ, unpublished data, [2012]) ) that reported the mRNA transcriptome in human patients of both genders belonging to either normal/ ICM/ HCM/ DCM with the information of gender for each sample (Table1). Each of these studies included both male and female samples. The probe set IDs of each data were converted into ENSEMBL gene ID, discarding those that failed to be converted. These data were then merged discarding probes that were not common to all these data. The final number of probes, common to all the studies integrated, was 11,942 and 12,954 in mouse and human, respectively. As for human, we discarded samples younger than 30. This effectively matched the mean and variance of age between male and female for each disease (S13 Fig). The batch effect of GSE22253 was removed by COMBAT method[1] as implemented in R sva package. Since COMBAT has been reported to outperform other normalization methods in cross-platform normalization[13,14], we adopted this method.. Differential expression analysisThe normalized data were analyzed using R (version 3.2) and the Bioconductor limma package[15]. Since Affymetrix GeneChip® miRNA 3.0 was based on the miRBase ver.17 and included probes for miRNAs excluded from the latest miRBase ver.21, these probes were first excluded from the following analyses. This procedure left 1,088 mouse mature miRNAs and 1,725 human mature miRNAs. Statistical analysis for assessing the differential gene expression was performed using the robust eBayes function to calculate moderated paired t-statistics after the linear model fit. All p-values were adjusted for false discovery rate correction (FDR < 0.05). For the downstream analysis to be based on the most reliable expression data, probe sets with signal intensity over certain threshold in at least one sample group (e.g. male ICM group) were included in the analysis. This threshold was set to 1 for miRNA and 4 for mRNA. Since cardiac sex difference presumably reflects the mild biases in transcriptome-wide gene expression, 1.2 fold change was considered meaningful. Hence our detection criteria was: FDR < 0.05 and fold change > 1.2.Clustering analysisSupport vector machine (SVM) and linear discriminant analysis (LDA) were conducted in R to assess if desired sample characteristics (i.e. male vs female / normal vs ICM vs HCM vs DCM) are discriminated based on the principal components of the transcriptome. Principal component analysis (PCA) was performed without scaling to reflect the fact that the data of the strongly expressed genes are more reliable. The number of principal components was chosen so that the following SVM or LDA discriminates the sample characteristics with minimum error. Error was estimated by leave-one-out cross-validation (LOOCV). P-value of this error was estimated by repeatedly (1000 times) testing the LOOCV error of the same model after randomly shuffling the sample labels (i.e. sex/ condition). SVM was adopted to discriminate male and female of the human miRNA array samples due to its superior discriminative capacity. Radial kernel was used to allow for non-linear hyperplane. Cost and gamma values were determined so that they minimize the misclassification error. LDA was adopted for the other discrimination tests for its superiority over SVM in computational cost and variability. SVM and LDA were conducted using e1071 package and MASS package in R, respectively.GO/pathway enrichment analysis of sexually dimorphic mRNAs/ sexually dimorphic mRNAs involved in sex-specific miRNA-mRNA networksWe checked the overrepresented GO/pathways in sexually dimorphic mRNAs/ sexually dimorphic mRNAs involved in sex-specific miRNA-mRNA networks using DAVID (Database for Annotation Visualization and Integrated Discovery, National Institute of Allergy and Infectious Disease)  GO/ pathway enrichment analysis software version 6.8 [16,17]. DAVID categorizes the genes into groups based on Gene Ontology/ pathway terms, and then displays similar annotations together. The annotation terms are clustered based on the share of common genes and an EASE score, a modified Fisher exact p-value, is produced for each annotation term. Next, the annotation terms were clustered based on similar annotation terms and genes. The overall enrichment score of each cluster was a minus log transformation of a geometric mean of all the EASE scores. Clusters with a minimum enrichment score of 1 (equivalent to a p-value of 0.1) were deemed significant. DAVID GO/ pathway enrichment analyses were performed with classification stringency of “high”. As the background, the total set of genes included in the merged meta-data was used.miRNA target pathway enrichment analysisWe constructed an in silico analysis pipeline wherein our male-female comparison result was input to the DIANA microT-CDS software[18,19] and the predicted targets were fetched, which were then input to the DAVID. Clusters with a minimum enrichment score of 1 (equivalent to a p-value of 0.1) were deemed significant. DAVID GO/ pathway enrichment analysis were performed with its default parameters to rule out the authors’ arbitrariness. As the background, the total set of genes included in the merged meta-data was used. If the DIANA microT-CDS software with default parameters returned > 3000 predicted target genes, which is the maximum number of input DAVID GO/ pathway cluster analysis can handle, we chose a prediction parameter more stringent than default (0.7) so that the total number of predicted target genes does not exceed 3000. For mouse sham/ MI heart, parameters were set to 0.72 and 0.86, respectively. For human normal/ ICM heart, parameters were set to 0.85 and 0.7, respectively.miRNA - mRNA network analysisFirst, lists of sexually dimorphic miRNAs and mRNAs were prepared along with the predicted targets of the sexually dimorphic miRNAs scanned from DIANA microT-CDS software. For this analysis we chose a DIANA microT-CDS prediction parameter of 0.5, which is less stringent than default (0.7) to increase the detection power. We implemented an algorithm such that if a sex mRNA, predicted to be targeted by any of the sex miRNAs, showed sex difference that anti-correlates with that of the sexually dimorphic miRNA targeting it, this mRNA – miRNA relationship was counted as “hit”. These “hits” were then visualized as a network in R using networkD3 package.Genomic loci enrichment analysismiRNAs and mRNAs’ genomic loci were obtained in R using biomaRt package[20,21]. The total number of miRNAs/ genes on each chromosome were calculated and normalized to 1. Same calculation was done for sex miRNAs/ genes, and the fold enrichment was calculated for each chromosome simply by dividing the normalized number of sex miRNAs/ genes by that of total miRNAs/ genes. For chromosomes that showed higher ratio in the sexually biased genes than in the total genes considered, statistical significance was assessed by randomly choosing miRNAs/ genes of the same number as sexually biased genes and calculating the fold enrichment of the chromosome of interest. This random sampling procedure was repeated for 1,000 times, and the p-value was estimated as the frequency of obtaining the fold enrichment larger than that in the actual data.miRNA RT-qPCRTotal RNA was extracted from sham and MI operated hearts using Sepasol (Sepasol-RNA I super G, nakalai tesque, Japan). cDNA synthesis and qRT-PCR were conducted using Mir-X miRNA First-Strand Synthesis Kit (Takara Clonetech). Briefly, microRNAs were polyadenylated and reverse transcribed with a universal primer. First strand cDNAs were amplified using a universal qPCR reverse primer with a forward primer designed to target the specific microRNA sequence of interest. Relative expression was evaluated by the delta Ct (cycle threshold) method, normalizing to mmu-miR-23a-3p, which showed the least variance in our microarray data (data not shown).We confirmed that even when mmu-let-7c-5p, another miRNA with low variance, was used as a reference, result did not change significantly (data not shown). The forward primers used are listed below.miRNA	Sequence	
mmu-miR-193a-5p	TGGGTCTTTGCGGGCAAGATGA	
mmu-miR-208b-3p	ATAAGACGAACAAAAGGTTTGT	
mmu-miR-23a-5p	GGGGTTCCTGGGGATGGGATTT	
mmu-miR-29b-3p	TAGCACCATTTGAAATCAGTGTT	
mmu-miR-30e-5p	TGTAAACATCCTTGACTGGAAG	
mmu-miR-3473a	TGGAGAGATGGCTCAGCA	
mmu-miR-505-5p	GGGAGCCAGGAAGTATTGATGTT	
mmu-miR-664-5p	CTGGCTGGGGAAAATGACTGG	
mmu-miR-709	GGAGGCAGAGGCAGGAGGA	
mmu-miR-744-5p	TGCGGGGCTAGGGCTAACAGCA	
mmu-miR-23a-3p	ATCACATTGCCAGGGATTTCC	
Data analysisP < 0.05 were considered significant. Multiple testing was corrected with a Benjamini–Hochberg procedure, as appropriate. All the data presented in this study were visualized in R using ggplot2 package unless specified otherwise.Supporting References1. 	Johnson WE, Li C, Rabinovic A. Adjusting batch effects in microarray expression data using empirical Bayes methods. Biostatistics. 2007;8: 118–127. doi:10.1093/biostatistics/kxj0372. 	R: a language and environment for statistical computing [Internet]. 2015 [cited 10 Nov 2015]. Available: http://www.gbif.org/resource/812873. 	Chen Q, Williams R, Healy CL, Wright CD, Wu SC, O’Connell TD. An association between gene expression and better survival in female mice following myocardial infarction. J Mol Cell Cardiol. Elsevier Ltd; 2010;49: 801–811. doi:10.1016/j.yjmcc.2010.08.0024. 	Kararigas G, Fliegner D, Gustafsson J-Å, Regitz-Zagrosek V. Role of the estrogen/estrogen-receptor-beta axis in the genomic response to pressure overload-induced hypertrophy. Physiol Genomics. 2011;43: 438–446. doi:10.1152/physiolgenomics.00199.20105. 	Fliegner D, Schubert C, Penkalla A, Witt H, Kararigas G, Dworatzek E, et al. Female sex and estrogen receptor- ␤attenuate cardiac remodeling and apoptosis in pressure overload. 2010; 1597–1606. doi:10.1152/ajpregu.00825.2009.6. 	Witt H, Schubert C, Jaekel J, Fliegner D, Penkalla A, Tiemann K, et al. Sex-specific pathways in early cardiac response to pressure overload in mice. J Mol Med. 2008;86: 1013–1024. doi:10.1007/s00109-008-0385-47. 	Coronado MJ, Brandt JE, Kim E, Bucek A, Bedja D, Abston ED, et al. Testosterone and interleukin-1 increase cardiac remodeling during coxsackievirus B3 myocarditis via serpin A 3n. AJP Hear Circ Physiol. 2012;302: H1726–H1736. doi:10.1152/ajpheart.00783.20118. 	Liu Y, Morley M, Brandimarto J, Hannenhalli S, Hu Y, Ashley E a., et al. RNA-Seq identifies novel myocardial gene expression signatures of heart failure. Genomics. 2015;105: 83–89. doi:10.1016/j.ygeno.2014.12.0029. 	Gaertner  a., Schwientek P, Ellinghaus P, Summer H, Golz S, Kassner  a., et al. Myocardial transcriptome analysis of human arrhythmogenic right ventricular cardiomyopathy. Physiol Genomics. 2012;44: 99–109. doi:10.1152/physiolgenomics.00094.201110. 	Pilbrow AP, Folkersen L, Pearson JF, Brown CM, McNoe L, Wang NM, et al. The chromosome 9p21.3 coronary heart disease risk allele is associated with altered gene expression in normal heart and vascular tissues. PLoS One. 2012;7: 1–11. doi:10.1371/journal.pone.003957411. 	Greco S, Fasanaro P, Castelvecchio S, D&apos;Alessandra Y, Arcelli D, Di Donato M, et al. MicroRNA dysregulation in diabetic ischemic heart failure patients. Diabetes. 2012;61: 1633–1641. doi:10.2337/db11-095212. 	Akat KM, Moore-McGriff D, Morozov P, Brown M, Gogakos T, Correa Da Rosa J, et al. Comparative RNA-sequencing analysis of myocardial and circulating small RNAs in human heart failure and their utility as biomarkers. Proc Natl Acad Sci U S A. 2014;111: 11151–6. doi:10.1073/pnas.140172411113. 	Rudy J, Valafar F. Empirical comparison of cross-platform normalization methods for gene expression data. BMC Bioinformatics. BioMed Central Ltd; 2011;12: 467. doi:10.1186/1471-2105-12-46714. 	Turnbull AK, Kitchen RR, Larionov A a, Renshaw L, Dixon J, Sims AH. Direct integration of intensity-level data from Affymetrix and Illumina microarrays improves statistical power for robust reanalysis. BMC Med Genomics. BMC Medical Genomics; 2012;5: 35. doi:10.1186/1755-8794-5-3515. 	Ritchie ME, Phipson B, Wu D, Hu Y, Law CW, Shi W, et al. limma powers differential expression analyses for RNA-sequencing and microarray studies. 2015;43. doi:10.1093/nar/gkv00716. 	Huang DW, Sherman BT, Lempicki R a. Systematic and integrative analysis of large gene lists using DAVID bioinformatics resources. Nat Protoc. 2009;4: 44–57. doi:10.1038/nprot.2008.21117. 	Huang DW, Sherman BT, Lempicki R a. Bioinformatics enrichment tools: Paths toward the comprehensive functional analysis of large gene lists. Nucleic Acids Res. 2009;37: 1–13. doi:10.1093/nar/gkn92318. 	Paraskevopoulou MD, Georgakilas G, Kostoulas N, Vlachos IS, Vergoulis T, Reczko M, et al. DIANA-microT web server v5.0: service integration into miRNA functional analysis workflows. Nucleic Acids Res. 2013;41: 169–173. doi:10.1093/nar/gkt39319. 	Reczko M, Maragkakis M, Alexiou P, Grosse I, Hatzigeorgiou  a G. Functional microRNA targets in protein coding sequences. Bioinformatics. 2012;28: 771–776. doi:10.1093/bioinformatics/bts043\rbts043 [pii]20. 	Durinck S, Spellman PT, Birney E, Huber W. Mapping identifiers for the integration of genomic datasets with the R/Bioconductor package biomaRt. Nat Protoc. 2009;4: 1184–1191. doi:10.1038/nprot.2009.9721. 	Durinck S, Moreau Y, Kasprzyk A, Davis S, De Moor B, Brazma A, et al. BioMart and Bioconductor: A powerful link between biological databases and microarray data analysis. Bioinformatics. 2005;21: 3439–3440. doi:10.1093/bioinformatics/bti525
